# Protein profiling by reverse phase protein array (RPPA) in classical hairy cell leukemia (HCL) and HCL‐variant

**DOI:** 10.1002/jha2.558

**Published:** 2022-09-02

**Authors:** Fieke W. Hoff, Ti'ara L. Griffen, Yihua Qiu, Steven M. Kornblau

**Affiliations:** ^1^ Department of Internal Medicine UT Southwestern Medical Center Dallas Texas USA; ^2^ Department of Microbiology, Biochemistry, and Immunology Morehouse School of Medicine Atlanta GA USA; ^3^ Department of Leukemia UT MD Anderson Cancer Center Houston Texas USA

**Keywords:** CLL, hairy cell leukaemia, leukemia

## Abstract

Classical hairy cell leukemia (HCL‐c) and HCL variant (HCL‐v) are recognized as separate entities with HCL‐v having significantly shorter overall survival. Proteomic studies, shown to be prognostic in various forms of leukemia, have not been performed in HCL. We performed reverse phase protein array‐based protein profiling with 384 antibodies in HCL‐c (*n* = 12), HCL‐v (*n* = 4), and normal B‐cells (*n* = 5) samples. While HCL could be distinguished from normal based on unsupervised hierarchical clustering, overlap in protein expression patterns was seen between HCL‐c and HCL‐v, with ∼10% of the proteins being differentially expressed, suggesting potential therapeutic targets.

## 1 INTRODUCTION

Hairy cell leukemia (HCL) is a rare, indolent B‐cell hematologic malignancy, comprising 2% of all leukemia cases [1], that is characterized by small mature B‐cells with abundant cytoplasm and “hairy” cell membrane projections. Immunophenotypically, HCL expresses B‐cell markers CD19, CD20, CD22, in combination with CD11c, CD25, CD103, CD123, annexin A1 and tartrate‐resistant acid phosphatase (TRAP). HCL arises from late mature memory B‐cells that acquire the BRAF‐V600E mutation, resulting in aberrant signaling through the RAF‐MEK‐ERK pathway [2].

The World Health Organization has recognized HCL‐variant (HCL‐v) as a separate entity from the classical HCL (HCL‐c) with morphological overlap, but distinguishing features, including lack of the BRAF V600E mutation, and absent expression of CD25, CD123, and TRAP. However, mutations in MAP2K1, coding for MEK1, a downstream protein of the RAF‐MEK‐ERK‐pathway are seen in half of HCL‐v, suggesting a pathophysiological similarity to HCL‐c mutations [3]. Although, HCL‐c responds well to apoptosis‐inducing purine analogs, HCL‐v is more aggressive and has poorer therapy responses. The 5‐year overall survival rates for HCL‐c and HCL‐v are approximately 95% and 60%, respectively.

The molecular landmarks of HCL‐v are much less known than these in HCL‐c, and the number of studies that directly compare HCL‐c to HCL‐v is limited. Hockley et al. performed genomic profiling in HCL‐c (*n* = 14) and HCL‐v (*n* = 15), finding deletion of 7q in both subtypes, while gains on chromosome 5 were more frequent in HCL‐v, and partial or complete deletion of 17p was limited to HCL‐v [4], suggesting that TP53 loss may contribute to greater genomic instability, aggressiveness and chemo‐refractoriness in HCL‐v. Others showed that while copy‐number alterations (CNAs) as well as copy neutral loss of heterozygosity were common in both HCL‐c and HCL‐v, HCL‐c lacked recurrent CNAs present in HCL‐v [5]. Proteomic profiling is predictive of prognosis in various lymphoid and myeloid malignancies but has not been studied in HCL. We aimed to compare relative protein expression patterns in HCL to normal CD19 cells, as well as to compare HCL‐c to HCL‐v with the goal of identifying differences in biology that point toward survival benefit, and given the lack of effective therapeutic strategies, identify proteins that may potentially aid in therapeutic optimization.

## 2 METHODS

### 2.1 Study population

Fresh peripheral blood (*n* = 12) and bone marrow aspirates (*n* = 4) were acquired from patients with HCL‐c (*n* = 12) and HCL‐v (*n* = 4) at MD Anderson Cancer Center (MDACC) between 2013 and 2018. All samples were collected within a year of diagnosis, before exposure to treatment, and collected and analyzed under MDACC Institutional Review Board protocol Lab01‐473, 2019‐0416, respectively. Informed consent was obtained in accordance with the Declaration of Helsinki. HCL‐c patients were treated with cladribine plus rituximab (*n* = 10), and two patients were never treated. Among the HCL‐v patients, one was treated with cladribine plus rituximab, one with ibrutinib, and two remained untreated. All patients were still alive at the end of follow‐up (median follow‐up, 4.8 years), one HCL‐v patient relapsed (Table S1).

### 2.2 Reverse phase protein array methodology

Reverse phase protein array (RPPA) was used to measure relative protein expression levels in 12 HCL‐c, four HCL‐v, and five normal CD19+ samples from healthy donors along with samples from patients with CLL and several other mature small B‐cell leukemias/lymphomas [6]. Lymphocytes were purified and processed to produce whole cell lysates as previously described [6, 7]. A total of 384 strictly validated primary antibodies was used (Table S2). Antibodies that had been previously validated were selected based on potential relevance to CLL, other leukemias, or other cancers. The median expression of CD19+ control samples was subtracted to normalize values to a normal median of zero enabling recognition of whether expression was similar to, above or below that of normal.

## 3 RESULTS AND DISCUSSION

**FIGURE 1:**
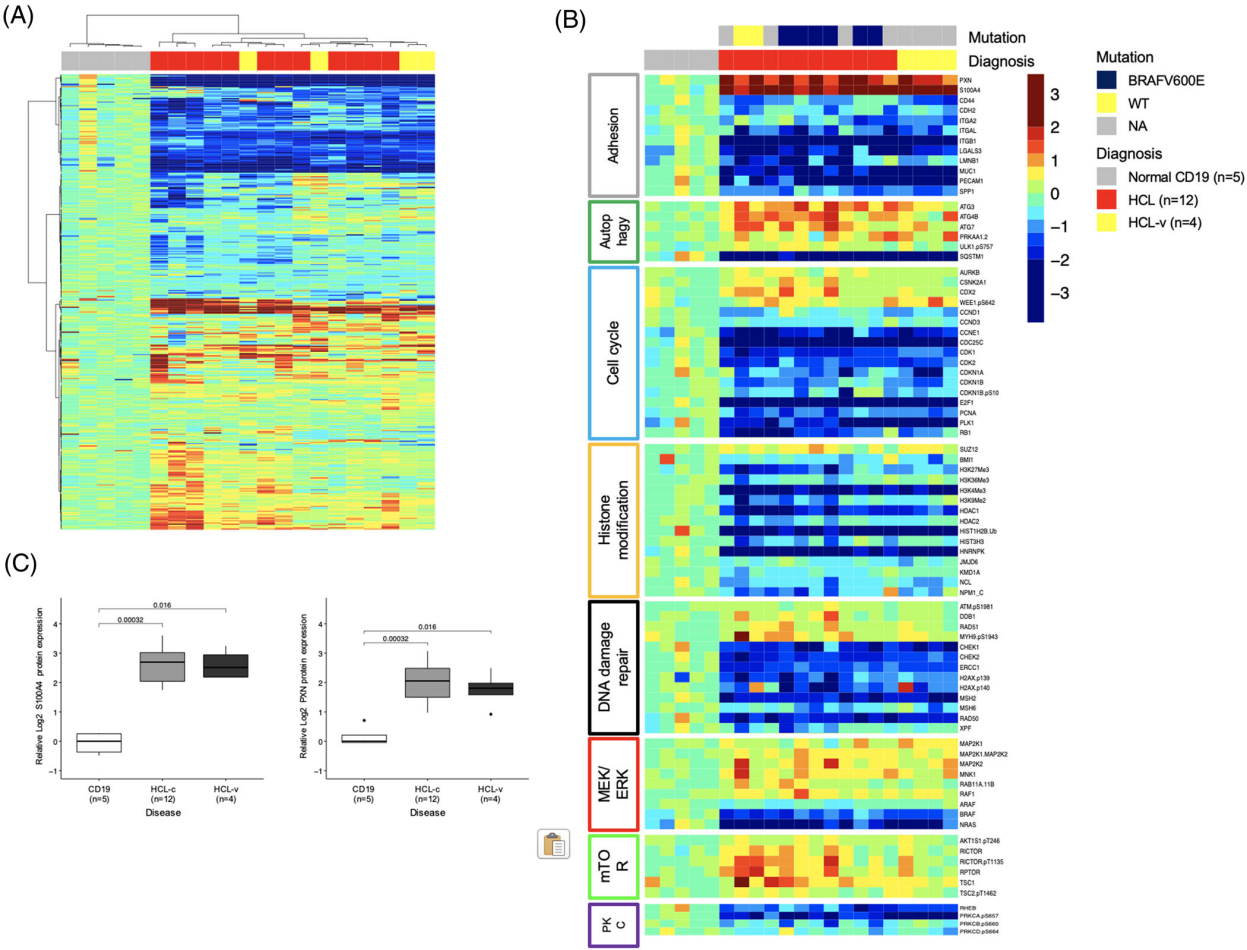
(A) Heatmap showing unsupervised hierarchical clustering of relative protein expression levels (*n* = 384 antibodies) in classical hairy cell leukemia (HCL‐c) (*n* = 12), HCL‐variant (HCL‐v) (*n* = 4), and normal CD19+ B‐cell (*n* = 5) patient samples. (B) Selection of proteins with a significant different expression between HCL‐c and HCL‐v, grouped by function, and the normal B‐cells. (C) Boxplots showing relative protein expression levels for S100A4 and PXN across the three subsets

Relative protein expression levels were measured for 384 proteins and protein modifications, and compared between the three subsets. Unsupervised hierarchical clustering showed complete separation of HCL from normal CD19+ samples, with 200 (52%) of the proteins being significantly differentially expressed between the HCL versus normal (false discovery rate adjusted *p*‐value < 0.05) (Figure 1A) (Table S3). As shown in Figure 1A, the four HCL‐v cases were not clearly separated from the HCL‐c cases. To search for differences that might explain the different response to purine nucleoside analogs, protein expression levels were directly compared between HCV‐c and HCL‐v, identifying 47 (12%) differentially expressed proteins (*p*‐value < 0.05) (Table S4). This is greater than 5%, the expected false positive rate for that *p*‐value. Compared to expression in normal CD19+ B cells, the expression of all three of ARAF, BRAF, and NRAS had lower expression in all cases of HCL‐c and HCL‐v. In contrast downstream MEK1, MEK2 and MNK1 were higher in HCL compared to normal (Figure 1B, red). Despite BRAFV600E mutation seen in the majority of the HCL‐c cases, BRAF‐pSer445 was not significantly different from normal, and HCL‐v even tended to have the highest phosphorylation levels. However, as BRAFV600E is an activating missense mutation in codon 600 of exon 15 (V600E) of BRAF gene, BRAF is likely still activated, therefore causing increased expression of MEK1/2 as seen here [2]. Given that HCL‐v is associated with MAP2K1/MEK1 mutation in half of the patients, this may explain increased expression of MEK1/2 in HCL‐v. And, while HCL‐v is not sensitive to BRAF‐inhibitors, it potentially explains the sensitivity to MEK‐inhibitors exhibited in vitro, and which are currently being tested in clinical trials.

Moreover, PKC signaling proteins were relatively low expressed in HCL samples (i.e., PRKCA‐pSer657, PRKCB‐pSer660, PRKCD‐pSer664 (Figure 1B, purple)), and vice versa, most mTOR‐signaling proteins were overexpressed, including AKT1S1, RICTOR‐(phospho), RPTOR, and TSC2‐(phospho), with lower RHEB expression (Figure 1B, green). Various proteins involved in cell cycle regulation were differentially expressed, suggesting a role for alteration in cell proliferation activity in HCLs (e.g., high Wee1, low CCND1/D3/E1, CDK1/2, E2F1, PLK1, RB1) (Figure 1B, blue). It is known that inactivating mutations of the cell cycle inhibitor CDKN1B (p27) are present in a subset of the HCL‐patient [8]. Recently, a DNA methylation study found specific methylation patterns in HCL, completely distinct from other B‐cell tumor entities [9]. Several genomic mutations in epigenetic regulators have been found in HCL, including KMT2C, KDM6A, CREBBP, and ARID1A; however the phenotypic consequences of these mutations are highly context dependent, and their functional consequences in HCL are unknown. In this dataset, a high proportion of proteins related to epigenetic modification (10 of 31) and to histone 3 protein level and marks (5 of 8 antibodies on the array) were significantly different from the normal CD19 controls, all being lower except for SUZ12 (Figure 1B, orange). The opposite was seen in our cohort of 795 CLL patient samples, with most of the histone modification proteins higher than normal.

**FIGURE 2:**
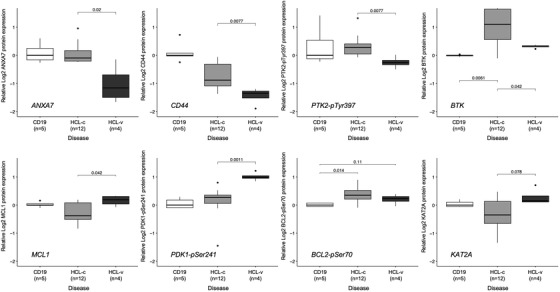
Boxplots showing relative proteins expression levels for ANXA7, CD44, PTK2‐pTyr397, BTK, MCL1, PDK1‐pSer241, BCL2‐pSer70, and KAT2A

Previously, gene expression profiling has revealed that HCL had significantly altered expression of genes involved in cell adhesion and response to chemokines, at least partially explaining the unique dissemination pattern of HCL [10]. We observed down regulation of various adhesion proteins when compared to normal (Figure 1B, grey). Particularly, PXN and S100A4 were markedly different, and S100A4 was previously identified as attractive target for cancer treatment (Figure 1C). Additionally, ANAX7, CD44, and PTK2‐pTyr397 were significantly lower expressed in HCL‐v compared to HCL‐c (Figure 2). Retrospective analyses identified higher PTK2 transcriptome levels as a favorable prognostic factor in CLL, and inhibition was associated with reduced rituximab‐dependent cell death in vitro [11]. PTK2 is a focal adhesion kinase that plays a key role in regulating cell migration, proliferation, and adhesion by integrating with the integrin signaling network. It is unclear how PTK2 exactly contributes to a favorable prognosis in CLL, but it is thought that rituximab induces cell death via homotypic adhesion.

In HCL‐c patients, Bruton's tyrosine kinase protein levels were heterogeneously expressed and significantly higher than normal; however, it was homogeneously lower in HCL‐v compared to HCL‐c (Figure 2). Previously, ibrutinib has been observed to induce responses more often in HCL‐c than HCL‐v, although updated results from the same study later showed no difference in outcome between HCL‐c and HCL‐v [12]. MCL1, often associated with resistance to treatment in other malignancies, had higher expression in HCL‐v as did PDK1‐pSer241, a kinase that activates substrates influencing a variety of cellular processes including proliferation, migration, and survival. In acute myeloid leukemia, PDK1 promotes survival through PKC activation [13]. We suggest that selectively targeting MCL1 in HCL‐v to counteract its antiapoptotic activity may be worthy of investigation for treatment in HCL‐v. Furthermore, BCL2‐Ser70 is known to confer resistance to drug‐induced apoptosis [14]. The observation that BCL2‐Ser70 was higher expressed in HCL supports the idea of targeting BCL2 to enhance drug‐induced cell death but needs to be further investigated. KAT2A was found to be an independent biomarker associated with renal cell carcinoma and prostate cancer with an epigenetic oncogenic role [15]. Although there are currently no KAT2A inhibitors available, inhibitors that target the upstream MCT1 are being explored in vivo. In contrast to what was previously published, we did not observe differences in TP53 protein expression levels.

In conclusion, this study uses proteomics for the first time in HCL‐c and HCL‐v. We found that protein expression patterns in HCL differed significantly from normal B‐cells and identified proteins that were differentially expressed between HCL‐c and HCL‐v. Larger studies with cell selection and multiomics, as well as dynamic pre‐post‐treatment measurements that could potentially allow early identification of increased relapse risk [16], are needed to further explore the molecular landscape of HCL. In particular, given the lack of high‐frequency mutations in HCL‐v, proteomics may be a useful tool to identify markers that point toward new treatment strategies.

## CONFLICT OF INTEREST

The authors declare they have no conflicts of interest.

### ETHICS STATEMENT

Written informed consent was provided according to the Declaration of Helsinki.

## Supporting information

Table S1. Patient characteristics.
Table S2. Rosetta Table of antibodies used for the RPPA
Table S3. Median expression levels of the HCL (*n* = 16) samples and the normal CD19 samples (*n* = 5), including uncorrected and FDR‐corrected *p*‐values. *p*‐Values were calculated using the Wilcoxon signed‐rank test.
Table S4. Median expression levels of the HCL‐c samples (*n* = 12) and the HCL‐v samples (*n* = 4), including uncorrected and FDR‐corrected *p*‐values. *p*‐values were calculated using the Wilcoxon signed‐rank test.

## Data Availability

The data that support the findings of this study are available from the corresponding author upon reasonable request.
