# Supplementary material for: Protein profiling by reverse phase protein array (RPPA) in classical hairy cell leukemia (HCL) and HCL‐variant
Source: EJHaem. 2022 Sep 2;3(4):1321–5. doi: 10.1002/jha2.558 (PMC9713071; doi:10.1002/jha2.558)
Supplement: Supplementary file 1 — Table S1. Patient characteristics. Table S2. Rosetta Table of antibodies used for the RPPA Table S3. Median expression levels of the HCL (n = 16) samples and the normal CD19 samples (n = 5), including uncorrected and FDR‐corrected p‐values. p‐Values were calculated using the Wilcoxon signed‐rank test. Table S4. Median expression levels of the HCL‐c samples (n = 12) and the HCL‐v samples (n = 4), including uncorrected and FDR‐corrected p‐values. p‐values were calculated using the Wilcoxon signed‐rank test. [file JHA2-3-1321-s001.pdf]

Supplementary Table S1. Patient characteristics (n=16)

**Characteristic**	**Variable**	**HCL-c**	**HCL-v**	**P**
Number	Count	12 (100)	4 (100)	
Age at diagnosis	Median (range)	58 (39-70)	63 (52-65)	0.464
Gender	Male	12 (100)	4 (100)	1.000
Ethnicity	Caucasian<br><br>African American<br><br>Unknown	10<br><br>0<br><br>2	3<br><br>1<br><br>0	0.2857
WBC (peripheral)	Median (range)	3.2 (1.3-20.2)	21.4 (15.4-23.3)	0.006
Absolute lymphocyte count	Median (range)	1.6 (0.3-6.5)	7.9 (4.0-12.8)	0.002
BRAF mutation	Mutated	7/7[†]/10	0/3	0.069
Outcome<br><br>Follow-up time (years)	Alive<br><br>Median (range)	12 (100%)<br><br>5.6 (0.1-8.7)	4 (100%)<br><br>2.4 (0.1-7.8)	

[†]Three patients lack BRAF mutation analysis as diagnosis preceded recognition of this in HCL

Supplementary Table S2

RPPA Antibody Name	HUGO Name	MIMI Name	Full.Name	Functional.Effect.of.Phosphorylation	Common.Name	Manufacturer	Catalog.Number	1st Antibody Dilution	Second Antibody Dilution	R2 RPPA.vs.WB	Host
ABL1	ABL1	ABL1	Tyrosine-protein kinase ABL1		Abl.C	Cell Signaling	2862	100	18000	>0.7	Rabbit
ACACA	ACACA	ACACA	Acetyl-CoA carboxylase 1		ACACA	Abcam	ab45174	2000	20000	0.5-0.7	Rabbit
ACACA.pS79	ACACA phospho serine 79	ACACA	Acetyl-CoA carboxylase 2	Inactivation	Acetyl.CoA.p	Cell Signaling	3661	10000	25000	>0.7	Rabbit
ACTB	ACTB	ACTB	Actin, cytoplasmic 1		Actin.beta	Sigma	A5441	10000	25000	0.5-0.7	mouse
AIFM1	AIFM1	AIFM1	Apoptosis-inducing factor 1, mitochondrial		AIF	Santa Cruz Biotechnology	sc13116	500	20000	0.5-0.7	mouse
AKR1C3	AKR1C3	AKR1C3	Aldo-keto reductase family 1 member C3		AKR1C3	marina.wang	marina.wang	1000	25000	0.5-0.7	mouse
AKT1	AKT1	AKT1	RAC-alpha serine/threonine-protein kinase		AKT1	Cell Signaling	2967	500	20000	>0.7	mouse
AKT1.2.3.pS473	AKT1 phospho serine 473	AKT1.AKT2.AKT3	RAC-alpha serine/threonine-protein kinase	Activation	AKT.p473	Cell Signaling	9271	150	25000	0.5-0.7	Rabbit
AKT1.2.3.pT308	AKT1/2/3 phospho threonine 308	AKT1.AKT2.AKT3	RAC-alpha serine/threonine-protein kinase	Activation	AKT.p308	Cell Signaling	9275	200	25000	0.5-0.7	Rabbit
AKT1.AKT2.AKT3	AKT1.AKT2.AKT3	AKT1.AKT2.AKT3	RAC-alpha serine/threonine-protein kinase		AKT	Cell Signaling	9272	250	20000	>0.7	Rabbit
AKT1S1.pT246	AKT1S1 phospho threonine 246	AKT1S1	Proline-rich AKT1 substrate 1	Inactivation	PRAS40.p	Cell Signaling	2997	100	20000	0.5-0.7	mouse
AKT2	AKT2	AKT2	RAC-beta serine/threonine-protein kinase		AKT2	Cell Signaling	2962	100	20000	>0.7	Rabbit
AKT3	AKT3	AKT3	RAC-gamma serine/threonine-protein kinase		akt3	Cell Signaling	4059	150	20000	>0.7	Rabbit
ANXA1	ANXA1	ANXA1	Annexin A1		ANXA1	BD Biosciences	610066	5000	25000	>0.7	mouse
ANXA7	ANXA7	ANXA7	Annexin A7		ANXA7	BD Biosciences	610668	250	18000	>0.7	mouse
ARAF	ARAF	ARAF	Serine/threonine-protein kinase A-Raf		Raf.A	Cell Signaling	4432	100	20000	>0.7	Rabbit
ARID1A	ARID1A	ARID1A	AT-rich interactive domain-containing protein 1A		ARID1A	Sigma	HPA005456	2000	20000	0.5-0.7	Rabbit
ASH2L	ASH2L	ASH2L	Set1/Ash2 histone methyltransferase complex subunit ASH2		Ash2L	Cell Signaling	5019	5000	20000	>0.7	Rabbit
ASNS	ASNS	ASNS	Asparagine synthetase		ASNS	Sigma	HPA029318	800	18000	0.5-0.7	Rabbit
ASS1	ASS1	ASS1	Argininosuccinate synthase		ASS1	Palars	palars	500	20000	0.5-0.7	mouse
ATF3	ATF3	ATF3	Cyclic AMP-dependent transcription factor ATF-3		ATF3	Abcam	ab87213	500	20000	>0.7	Rabbit
ATG3	ATG3	ATG3	Ubiquitin-like-conjugating enzyme ATG3		Atg3	Cell Signaling	3415	800	20000	>0.7	Rabbit
ATG4B	ATG4B	ATG4B	Cysteine protease ATG4B		Atg4B	cell signaling	13507	200	NA	0.5-0.7	Rabbit
ATG7	ATG7	ATG7	Ubiquitin-like modifier-activating enzyme ATG7		Atg7	Cell Signaling	8558	500	20000	>0.7	Rabbit
ATM	ATM	ATM	Serine-protein kinase ATM		ATM	Cell Signaling	2873	750	20000	>0.7	Rabbit
ATM.pS1981	ATM phospho serine 1981	ATM	Serine-protein kinase ATM	inactivation	ATM.p1981	Cell Signaling	5883	150	20000	>0.7	Rabbit
AURKB	AURKB	AURKB	Aurora kinase B		Aurora	Cell Signaling	3094	75	20000	>0.7	Rabbit
AXL	AXL	AXL	Tyrosine-protein kinase receptor UFO		AXL	Cell Signaling	8661	500	20000	>0.7	Rabbit
BAK	BAK	BAK	Bcl-2 homologous antagonist/killer		BAK	Abcam	ab32371	50	20000	0.5-0.7	Rabbit
BAX	BAX	BAX	Apoptosis regulator BAX		BAX	Cell Signaling	2772	300	18000	>0.7	Rabbit
BBC3	BBC3	BBC3	Bcl-2-binding component 3		puma	Cell Signaling	4976	100	20000	0.5-0.7	Rabbit
BCL2	BCL2	BCL2	Apoptosis regulator Bcl-2		Bcl2	CST	M0887	500	10000	>0.7	Rabbit
BCL2.pS70	BCL2 phospho serine 70	BCL2	Apoptosis regulator Bcl-3	activity inhibition	Bcl2.p70	Cell Signaling	2827	250	10000	0.5-0.7	Rabbit
BCL2A1	BCL2A1	BCL2A1	Bcl-2-related protein A1		BCL2A1	abnova	PAB8528	2000	25000	>0.7	Rabbit
BCL2L1	BCL2L1	BCL2L1	Bcl-2-like protein 1		Bxl.XL	Cell Signaling	2762	400	18000	>0.7	Rabbit
BCL2L11	BCL2L11	BCL2L11	Bcl-2-like protein 11		Bim	Abcam	ab32158	50	15000	>0.7	Rabbit
BECN1	BECN1	BECN1	Beclin-1		beclin.1	Cell Signaling	3738	400	20000	>0.7	Rabbit
BID	BID	BID	BH3-interacting domain death agonist		Bid	Cell Signaling	2002	500	20000	0.5-0.7	Rabbit
BIRC2	BIRC2	BIRC2	Baculoviral IAP repeat-containing protein 2		CIAP.1	upstate	7.759	500	20000	0.5-0.7	Rabbit
BIRC3	BIRC3	BIRC3	Baculoviral IAP repeat-containing protein 3		CIAP2	Cell Signaling	3130	400	18000	0.5-0.7	Rabbit
BIRC5	BIRC5	BIRC5	Baculoviral IAP repeat containing 5		survivin	Abcam	ab76424	1000	25000	>0.7	Rabbit
BMI1	BMI1	BMI1	Polycomb complex protein BMI-1		Bmi.1	Cell Signaling	2830	50	NA	0.5-0.7	Rabbit
BRAF	BRAF	BRAF	Serine/threonine-protein kinase B-raf		Raf.B	Santa Cruz Biotechnology	sc5284	100	20000	0.5-0.7	mouse
BRAF.pS445	BRAF phospho serine 445	BRAF	Serine/threonine-protein kinase B-raf	Activation	Raf.B.p	Cell Signaling	2696	300	20000	>0.7	Rabbit
BRCA2	BRCA2	BRCA2	Breast cancer type 2 susceptibility protein		BRCA2	Cell Signaling	9012	75	20000	0.5-0.7	Rabbit
BRD4	BRD4	BRD4	Bromodomain-containing protein 4		BRD4	epitomics	5716.1	1000	20000	0.5-0.7	Rabbit
BTK	BTK	BTK	Tyrosine-protein kinase BTK		BTK	Abclonal	A19002	300	10000	0.579	Rabbit
CASP3	CASP3	CASP3	Fructose-2,6-bisphosphatase TIGAR		Caspase3	Cell Signaling	9662	1000	20000	0.5-0.7	Rabbit
CASP3_cleaved	CASP3 cleaved	CASP3	Caspase-3		caspase3.cle	Abcam	ab32042	1000	25000	0.5-0.7	Rabbit
CASP7_cleaved	CASP7 cleaved	CASP7	Caspase-7		Caspase7.cle	Cell Signaling	9491	75	25000	0.5-0.7	Rabbit
CASP9	CASP9	CASP9	Caspase-9		caspase9	BD PharMingen	551246	100	20000	0.5-0.7	mouse
CAV1	CAV1	CAV1	Caveolin-1		caveolin.1	Cell Signaling	3238	75	20000	>0.7	Rabbit
CBL	CBL	CBL	E3 ubiquitin-protein ligase CBL		cbl.c	BD Transduction Lab	610449	250	15000	0.6532	mouse
CBX7	CBX7	CBX7	Chromobox protein homolog 7		cbx7	Abcam	ab21873	1000	18000	0.5921	Rabbit
CCNB1	CCNB1	CCNB1	G2/mitotic-specific cyclin-B1		CyclinB1	Epitomics	1495.1	1000	25000	>0.7	Rabbit
CCND1	CCND1	CCND1	G1/S-specific cyclin-D1		cyclinD1	Santa Cruz Biotechnology	sc718	200	20000	>0.7	Rabbit
CCND3	CCND3	CCND3	G1/S-specific cyclin-D3		cyclinD3	Cell Signaling	2936	200	20000	0.5-0.7	mouse
CCNE1	CCNE1	CCNE1	G1/S-specific cyclin-E1		cyclinE1	Santa Cruz Biotechnology	sc247	100	20000	>0.7	mouse
CD276	CD276	CD276	CD276 antigen		B7.H3	cell signaling	14058	50	18000	0.5-0.7	Rabbit
CD4	CD4	CD4	T-cell surface glycoprotein CD4		CD4	Abcam	ab133616	500	18000	>0.7	Rabbit
CD44	CD44	CD44	CD44 antigen		CD44	Cell Signaling	3570	100	20000	0.5-0.7	mouse
CD74	CD74	CD74	HLA class II histocompatibility antigen gamma chain		CD74	Santa Cruz Biotechnology	sc6262	75	20000	0.5-0.7	mouse
CD86	CD86	CD86	T-lymphocyte activation antigen CD86		CD86	Abcam	ab53004	2000	20000	>0.7	Rabbit
CDC25C	CDC25C	CDC25C	M-phase inducer phosphatase 3		CDC25C	Cell Signaling	4688	200	18000	>0.7	Rabbit
CDH1	CDH1	CDH1	Cadherin-1		CadherinE	Cell Signaling	3195	50	15000	>0.7	Rabbit
CDH2	CDH2	CDH2	Cadherin-2		CadherinN	Cell Signaling	4061	50	15000	>0.7	Rabbit
CDK1	CDK1	CDK1	Cyclin-dependent kinase 1		cdc2	calbiochem	cc01	75	25000	0.5-0.7	mouse
CDK1.pY15	CDK1 phospho tyrosine 15	CDK1	Cyclin-dependent kinase 1	inactivation	CDC2.p15	Cell Signaling	4539	250	20000	0.5-0.7	Rabbit
CDK2	CDK2	CDK2	Cyclin-dependent kinase 2		Cdk2	Santa Cruz Biotechnology	sc6248	25	15000	>0.7	mouse
CDKN1A	CDKN1A	CDKN1A	Cyclin-dependent kinase inhibitor 1		P21	Santa Cruz Biotechnology	sc397	1000	NA	0.5-0.7	Rabbit
CDKN1B	CDKN1B	CDKN1B	Cyclin-dependent kinase inhibitor 1B		P27	Abcam	ab32034	100	20000	>0.7	Rabbit
CDKN1B.pS10	CDKN1B phospho serine 10	CDKN1B	Cyclin-dependent kinase inhibitor 1B	Apoptosis altered	P27.p10	Epitomics	2187.1	500	20000	>0.7	Rabbit
CDKN1B.pT157	CDKN1B phospho threonine 157	CDKN1B	Cyclin-dependent kinase inhibitor 1B	Apoptosis altered	P27.p157	R&D System	AF1555	200	20000	0.5-0.7	Rabbit
CDKN1B.pT198	CDKN1B phospho threonine 198	CDKN1B	Cyclin-dependent kinase inhibitor 1B	Cell growth induced	P27.p198	Abcam	ab64949	800	20000	>0.7	Rabbit
CDKN2A	CDKN2A	CDKN2A	Cyclin-dependent kinase inhibitor 2A		P16	Abcam	ab81278	2000	25000	>0.7	Rabbit
CDX2	CDX2	CDX2	Homeobox protein CDX-2		CDX2	cell signaling	12306	25	15000	0.5-0.7	Rabbit
CHEK1	CHEK1	CHEK1	Serine/threonine-protein kinase Chk1		CHK1	Cell Signaling	2360	250	18000	0.5-0.7	mouse
CHEK1.pS296	CHEK1 phospho serine 296	CHEK1	Serine/threonine-protein kinase Chk1	Activation	CHK1.p296	Abcam	ab79758	150	20000	>0.7	Rabbit
CHEK1.pS345	CHEK1 phospho serine 296	CHEK1	Serine/threonine-protein kinase Chk1	enzymatic activity	CHK1.p345	Cell Signaling	2348	250	25000	0.5-0.7	Rabbit
CHEK2	CHEK2	CHEK2	Serine/threonine-protein kinase Chk2		CHK2	Cell Signaling	3440	150	20000	>0.7	mouse
CHEK2.pT68	CHEK2 phospho threonine 68	CHEK2	Serine/threonine-protein kinase Chk2	enzymatic activity	CHK2.p68	Cell Signaling	2197	500	25000	0.5-0.7	Rabbit

CLPP	CLPP	CLPP	ATP-dependent Clp protease proteolytic subunit, mitochondrial		CLPP	Abcam	ab124822	500	20000	>0.7	Rabbit
COG3	COG3	COG3	Conserved oligomeric Golgi complex subunit 3		COG3	protein Tech	11130.1.AP	2000	20000	>0.7	Rabbit
COPS5	COPS5	COPS5	COP9 signalosome complex subunit 5		JAB1	Santa Cruz Biotechnology	sc13157	400	15000	>0.7	mouse
CREB1	CREB1	CREB1	Cyclic AMP-responsive element-binding protein 1		CREB	Epitomics	1496.1	2000	18000	>0.7	Rabbit
CREB1.pS133	CREB1 phospho serine 133	CREB1	Cyclic AMP-responsive element-binding protein 1	Activation	CREB.p133	Epitomics	1113.1	2000	18000	0.5-0.7	Rabbit
CSNK2A1	CSNK2A1	CSNK2A1	Casein kinase II subunit alpha		CK.alpha2	Cell Signaling	2656	100	20000	0.5-0.7	Rabbit
CTNNB1	CTNNB1	CTNNB1	Catenin beta-1		catenin.beta	Cell Signaling	9562	100	20000	>0.7	Rabbit
CTNNB1.pS33.S37.T41	CTNNB1 phospho serine 33,37,threonine 41	CTNNB1	Catenin beta-1	cell adhesion/growth altered	catenin.beta.p33.37.41	Cell Signaling	9561	2000	20000	>0.7	Rabbit
CTNNB1.pT41.S45	CTNNB1 phospho threonine 41,45	CTNNB1	Catenin beta-1	cell adhesion/growth altered	catenin.p41.45	Cell Signaling	9565	250	20000	>0.7	Rabbit
CTSG	CTSG	CTSG	Cathepsin G		cathepsinG	Abcam	ab8816	500	30000	>0.7	sheep
CXCR5	CXCR5	CXCR5	C-X-C chemokine receptor type 5		CXCR5	Abcam	EPR8837	500	10000	0.52	Rabbit
DDB1	DDB1	DDB1	DNA damage-binding protein 1		DDB.1	Cell Signaling	6998	800	18000	>0.7	Rabbit
DDX17	DDX17	DDX17	Probable ATP-dependent RNA helicase DDX17		DDX17	Abcam	ab180190	2000	15000	0.5-0.7	rabbit
DLX1	DLX1	DLX1	Homeobox protein DLX-1		DLX1	abnova	H00001745.M01	2000	18000	0.5-0.7	mouse
DNM1L	DNM1L	DNM1L	Dynamin-1-like protein		DRP1	Cell Signaling	5391	1000	18000	>0.7	Rabbit
DNMT1	DNMT1	DNMT1	DNA (cytosine-5)-methyltransferase 1		DNMT1	Cell Signaling	5032	500	18000	>0.7	Rabbit
DUSP4	DUSP4	DUSP4	Dual specificity protein phosphatase 4		DUSP4	Cell Signaling	5149	200	18000	>0.7	rabbit
DUSP6	DUSP6	DUSP6	Dual specificity protein phosphatase 6		DUSP6	Abcam	ab76310	5000	25000	>0.7	rabbit
DVL3	DVL3	DVL3	Segment polarity protein dishevelled homolog DVL-3		DVl3	Cell Signaling	3218	400	18000	>0.7	Rabbit
E2F1	E2F1	E2F1	Transcription factor E2F1		E2F.1	Santa Cruz Biotechnology	sc251	50	18000	>0.7	mouse
EEF2	EEF2	EEF2	Elongation factor 2		eEF2	Cell Signaling	2332	150	18000	0.5-0.7	Rabbit
EEF2K	EEF2K	EEF2K	Eukaryotic elongation factor 2 kinase		eEF2K	Cell Signaling	3692	75	20000	>0.7	Rabbit
EGFR	EGFR	EGFR	Epidermal growth factor receptor		EGFR	Cell Signaling	2232	75	20000	>0.7	Rabbit
EGFR.pY1173	EGFR phospho tyrosine 1173	EGFR	Epidermal growth factor receptor		EGFR.p	Abcam	ab32578	150	18000	>0.7	Rabbit
EGLN1	EGLN1	EGLN1	Egl nine homolog 1		Egln1	Millipore	5.1327	200	20000	0.5-0.7	mouse
EIF2AK2	EIF2AK2	EIF2AK2	Interferon-induced, double-stranded RNA-activated protein kinase		PRKR	Abnova	H0000561.M01	5000	18000	0.5-0.7	mouse
EIF2S1	EIF2S1	EIF2S1	Eukaryotic translation initiation factor 2 subunit 1		eIF2.alpha	Cell Signaling	9722	4000	18000	0.5-0.7	Rabbit
EIF2S1.pS51	EIF2S1 phospho serine 51	EIF2S1	Eukaryotic translation initiation factor 2 subunit 1	stabelizes	eIF2.alpha.p	Cell Signaling	9721	500	18000	>0.7	Rabbit
EIF4E	EIF4E	EIF4E	Eukaryotic translation initiation factor 4E		eIF4E	Cell Signaling	9742	800	18000	>0.7	Rabbit
EIF4E.pS209	EIF4E phospho serine 209	EIF4E	Eukaryotic translation initiation factor 4E	Activation	EIF4E.P209	Abcam	ab76256	1000	18000	>0.7	Rabbit
EIF4EBP1	EIF4EBP1	EIF4EBP1	Eukaryotic translation initiation factor 4E-binding protein 1		X4EBP1	Cell Signaling	9452	1000	18000	>0.7	Rabbit
EIF4EBP1.pT37.T46	EIF4EBP1 phospho threonine 37,41	EIF4EBP1	Eukaryotic translation initiation factor 4E-binding protein 1	Inactivation	X4EBP1.p37.46	Cell Signaling	9456	20000	20000	>0.7	Rabbit
EIF4G1	EIF4G1	EIF4G1	Eukaryotic translation initiation factor 4 gamma 1		EIF4G	Cell Signaling	2498	2000	20000	0.5-0.7	Rabbit
EIF4G2	EIF4G2	EIF4G2	Eukaryotic translation initiation factor 4 gamma 2		DAP5	BD Biosciences	610743	250	15000	>0.7	mouse
ELAVL1	ELAVL1	ELAVL1	ELAV-like protein 1		HuR	millipore	7.1735	2000	20000	>0.7	Rabbit
ELK1.pS383	ELK1 phospho serine 383	ELK1	ETS domain-containing protein Elk-1	Activation	EIK.1.p	Cell Signaling	9181	200	15000	0.5-0.7	Rabbit
EP300	EP300	EP300	Histone acetyltransferase p300		P300	Abcam	ab3164	250	18000	>0.7	mouse
EPHA2	EPHA2	EPHA2	Ephrin type-A receptor 2		EphRA2	Cell Signaling	6997	500	NA	>0.7	Rabbit
EPHA2.pS897	EPHA2 phospho serine 897	EPHA2	Ephrin type-A receptor 2	carcinogenesis induced	EphA2.p897	Cell Signaling	6347	400	NA	0.5-0.7	Rabbit
EPHA2.pY588	EPHA2 phospho tyrosine 588	EPHA2	Ephrin type-A receptor 2	cell adhesion altered	EphA2.p588	Cell signaling	12677	500	NA	0.5-0.7	Rabbit
ERBB2	ERBB2	ERBB2	Receptor tyrosine-protein kinase erbB-2		Her2	Lab Vision (fisher)	MS.325.p0	1000	20000	>0.7	mouse
ERBB2.pY1248	ERBB2 phospho tyrosine 1248	ERBB2	Receptor tyrosine-protein kinase erbB-2	Activation	Her2.p	R&D System	AF1768	2000	20000	0.5-0.7	Rabbit
ERCC1	ERCC1	ERCC1	DNA excision repair protein ERCC-1		ERCC1	Santa Cruz Biotechnology	sc17809	75	18000	>0.7	mouse
ERCC5	ERCC5	ERCC5	DNA repair protein complementing XP-G cells		ERCC5	protein Tech	11331.1.AP	750	20000	0.5-0.7	Rabbit
ERG	ERG	ERG	Transcriptional regulator ERG		Erg1.2.3	Santa Cruz Biotechnology	sc353	1000	20000	>0.7	Rabbit
ERN1	ERN1	ERN1	Serine/threonine-protein kinase/endoribonuclease IRE1		IRE1	Cell Signaling	3294	500	18000	0.5-0.7	Rabbit
ETS1	ETS1	ETS1	Protein C-ets-1		ETS.1	bethyl Lab	A303.501A	2000	20000	>0.7	Rabbit
EZH2	EZH2	EZH2	Histone-lysine N-methyltransferase EZH2		Ezh2	Cell Signaling	5246	1000	18000	>0.7	Rabbit
EZRIN	EZRIN	EZRIN	Ezrin		EZRIN	Abclonal	A19048	500	10000	0.611	Rabbit
FASN	FASN	FASN	Fatty acid synthase		FASN	Cell Signaling	3180	1000	20000	>0.7	Rabbit
FLI1	FLI1	FLI1	Friend leukemia integration 1 transcription factor		Fli	watson	watson	2000	20000	0.5-0.7	Rabbit
FN1	FN1	FN1	Fibronectin type III domain containing		FN1	Epitomics	1574.1	20000	15000	>0.7	Rabbit
FOXM1	FOXM1	FOXM1	Forkhead box protein M1		FoxM1	Cell Signaling	5436	75	18000	>0.7	Rabbit
FOXO3	FOXO3	FOXO3	Forkhead box protein O3		Foxo3.alpha	Cell Signaling	2497	50	15000	0.5-0.7	Rabbit
FOXO3.pS318.S321	FOXO3 phospho serine 318,321	FOXO3	Forkhead box protein O3	Inactivation	FOXO3.p	Cell Signaling	9465	10000	20000	0.5-0.7	Rabbit
FZR1	FZR1	FZR1	Fizzy-related protein homolog		FZR1	LS Bio	54697	500	20000	>0.7	Rabbit
G6PD	G6PD	G6PD	Glucose-6-phosphate 1-dehydrogenase		G6PD	Cell Signaling	8866	100	NA	>0.7	Rabbit
GAB2	GAB2	GAB2	GRB2-associated-binding protein 2		Gab2	Cell Signaling	3239	800	18000	>0.7	Rabbit
GAB2.pY452	GAB2 phospho tyrosine 452	GAB2	GRB2-associated-binding protein 2	molecular regulation	Gab2.p	Cell Signaling	3882	25	15000	0.5-0.7	Rabbit
GAPDH	GAPDH	GAPDH	Glyceraldehyde-3-phosphate dehydrogenase		GAPDH	Ambion	AM4300	5000	20000	0.5-0.7	mouse
GATA1	GATA1	GATA1	Erythroid transcription factor		GATA.1	Cell Signaling	3535	500	20000	>0.7	Rabbit
GATA3	GATA3	GATA3	Trans-acting T-cell-specific transcription factor GATA-3		GATA3	BD Biosciences	558686	1000	15000	>0.7	mouse
GLS	GLS	GLS	Glutaminase kidney isoform, mitochondrial		GLS	Abcam	ab156876	400	20000	0.5-0.7	Rabbit
GLUD	GLUD	GLUD	Glutamate dehydrogenase 1, mitochondrial		GLUD	cell signaling	12793	800	20000	0.5-0.7	Rabbit
GSK3A.B	GSK3A.B	GSK3A.B	Glycogen synthase kinase-3 alpha		GSK3	Santa Cruz Biotechnology	sc.7291	500	20000	>0.7	mouse
GSK3A.B.pS21.9	GSK3A.B phospho serine 21,9	GSK3A	Glycogen synthase kinase-3 beta	Inactivation	GSK.alpha.beta	Cell Signaling	9331	200	NA	>0.7	Rabbit
GYS1	GYS1	GYS1	Glycogen [starch] synthase, muscle		GYS1	Cell Signaling	3886	1000	20000	>0.7	Rabbit
GYS1.pS641	GYS1 phospho serine 641	GYS1	Glycogen [starch] synthase, muscle	Inactivation	GYS1.p	Cell Signaling	3891	400	20000	>0.7	Rabbit
H2AX.pS139	H2AX phospho serine 139	H2AX	Histone H2AX	DNA repair/apoptosis altered	H2AX.p139	e Biotechnology(Thermo Fishe	LFPA0025	1000	18000	0.5-0.7	Rabbit
H2AX.pS140	H2AX phospho serine 140	H2AX	Histone H2AX	cell cycle,growth,transcription inhibited	H2AX.p140	Abcam	MA1.2022	1000	NA	>0.7	mouse
H3K27Me3	HISTH3-K27-Me3	H3K27Me3	Trimethylated HistoneH3 lysine 27	Repression	H3K27Me3	active motif	61017	3000	20000	>0.7	mouse
H3K36Me3	HISTH3-K36-Me3	H3K36Me3	Trimethylated HistoneH3 lysine 36	Activation	H3K36Me3	active motif	61011	20000	20000	>0.7	Rabbit
H3K4Me1	HISTH3-K4-Me1	H3K4Me1	Monomethylated HistoneH3 lysine 4	Activation	H3K4Me1	Cell Signaling	5326	1000	18000	>0.7	Rabbit
H3K4Me2	HISTH3-K4-Me2	H3K4Me2	Dimethylated HistoneH3 lysine 4	Repression	H3K4Me2	active motif	39141	8000	18000	0.5-0.7	Rabbit
H3K4Me3	HISTH3-K4-Me3	H3K4Me3	Trimethylated HistoneH3 lysine 4	Activation	H3K4Me3	active motif	39159	10000	18000	0.5-0.7	Rabbit
H3K9Me2	HISTH3-K9-Me2	H3K9Me2	Dimethylated HistoneH3 lysine 9	Repression	H3K9Me2	Abcam	ab32521	2000	NA	>0.7	mouse
HDAC1	HDAC1	HDAC1	Histone deacetylase 1		HDAC1	Imgenex	IM.337	400	20000	0.5-0.7	Rabbit
HDAC2	HDAC2	HDAC2	Histone deacetylase 2		HDAC2	Santa Cruz Biotechnology	sc7899	1000	20000	>0.7	Rabbit
HDAC3	HDAC3	HDAC3	Histone deacetylase 3		HDAC3	Cell Signaling	2632	75	20000	0.5-0.7	Rabbit
HDAC6	HDAC6	HDAC6	Histone deacetylase 6		HDAC6	Cell Signaling	7558	10000	18000	>0.7	Rabbit
HES1	HES1	HES1	Transcription factor HES-1		HES1	cell signaling	11988	400	18000	>0.7	Rabbit
HEXIM1	HEXIM1	HEXIM1	Protein HEXIM1		HEXIM1	cell signaling	12604	1000	15000	>0.7	Rabbit
HIF1A	HIF1A	HIF1A	Hypoxia-inducible factor 1-alpha		HIF.1.alpha	BD pharMingen	610959	25	15000	0.5-0.7	Mouse
HIST1H2B.Ub	HIST1H2B.Ub	HIST1H2B.Ub	Histone H2B type 1-B		Ubiquityl.his.h2B	millipore	5.1312	50	18000	0.5-0.7	mouse

HIST3H3	HIST3H3	HIST3H3	Histone H3.1t		H3.Histone	active motif	39163	10000	20000	>0.7	Rabbit
HK2	HK2	HK2	Hexokinase-2		HexokinaseII	Cell Signaling	2867	75	18000	>0.7	Rabbit
HNRNPK	HNRNPK	HNRNPK	Heterogeneous nuclear ribonucleoprotein K		hnRNPK	Santa Cruz Biotechnology	sc28380	10000	18000	0.5-0.7	Mouse
HSF1	HSF1	HSF1	Heat shock factor protein 1		HSF1	Cell Signaling	4356	1000	20000	>0.7	Rabbit
HSF1.pS326	HSF1 phospho serine 326	HSF1	Heat shock factor protein 1	Activation	HSF1.p	Abcam	ab76076	10000	20000	>0.7	Rabbit
HSP90AA1.HSP90AB1	HSP90AA1.HSP90AB1	HSP90AA1.HSP90AB1	Heat shock protein HSP 90-alpha/beta		HSP90	Cell Signaling	4874	1000	18000	0.5-0.7	Rabbit
HSPA1A	HSPA1A	HSPA1A	Heat shock 70 kDa protein 1A		HSP70	Cell Signaling	4872	100	20000	0.5-0.7	Rabbit
HSPA5	HSPA5	HSPA5	78 kDa glucose-regulated protein		bip.GRP78	BD transduction laboraties	610978	1000	20000	>0.7	mouse
HSPA9	HSPA9	HSPA9	Stress-70 protein, mitochondrial		Grp75	Cell Signaling	3593	1000	18000	0.5-0.7	Rabbit
HSPB1.pS82	HSPB1 phospho serine 82	HSPB1	Heat shock protein beta-1		HSP27.p	Cell Signaling	2401	200	20000	>0.7	Rabbit
HSPD1	HSPD1	HSPD1	60 kDa heat shock protein, mitochondrial		HSP60	Cell Signaling	12165	1000	NA	>0.7	Rabbit
IGF1R.pT1135.T1136	IGF1R phospho threonine 1135, 1136	IGF1R	Insulin-like growth factor 1 receptor		IGF1R.p	Cell Signaling	3024	50	15000	>0.7	Rabbit
IGFBP2	IGFBP2	IGFBP2	Insulin-like growth factor-binding protein 2		IGFBP.2	Cell Signaling	3922	150	18000	>0.7	Rabbit
IGFR1	IGFR1	IGFR1	insulin like growth factor 1 receptor		IGFR.beta	Cell Signaling	3027	750	18000	0.5-0.7	Rabbit
INPPL1	INPPL1	INPPL1	Phosphatidylinositol 3,4,5-trisphosphate 5-phosphatase 2		SHIP2	Cell Signaling	2730	100	18000	>0.7	Rabbit
ITGA2	ITGA2	ITGA2	Integrin alpha-2		CD49b	sduction Lab) to BD bioscienc	611016	100	15000	>0.7	mouse
ITGAL	ITGAL	ITGAL	Integrin alpha-L		CD11a	BD Transduction Lab	610826	500	20000	>0.7	mouse
ITGB1	ITGB1	ITGB1	Integrin beta-1		CD29	BD Sciences	610467	75	15000	>0.7	mouse
JAG1	JAG1	JAG1	Protein jagged-1		jagged1	Abcam	ab109536	300	25000	>0.7	Rabbit
JAK2	JAK2	JAK2	Tyrosine-protein kinase JAK2		Jak2	Cell Signaling	3230	2000	25000	>0.7	Rabbit
JMJD6	JMJD6	JMJD6	Bifunctional arginine demethylase and lysyl-hydroxylase JMJD6		JMJD6	Abcam	ab50720	1000	20000	0.5-0.7	Rabbit
JUN.pS73	JUN phospho serine 73	JUN	Transcription factor AP-1	Activation	JunC.p	Cell Signaling	9164	NA	20000	>0.7	Rabbit
JUNB	JUNB	JUNB	Transcription factor jun-B		Jun.B	Cell Signaling	3755	100	18000	>0.7	Rabbit
KAT2A	KAT2A	KAT2A	Histone acetyltransferase KAT2A		GCN5L2	Cell Signaling	3305	200	18000	>0.7	Rabbit
KDR	KDR	KDR	Vascular endothelial growth factor receptor 2		VEGFR2	Cell Signaling	2479	500	20000	>0.7	Rabbit
KDR.pY1175	KDR phospho tyrosine 1175	KDR	Vascular endothelial growth factor receptor 2	cell growth altered	VEGFR2.p	Cell Signaling	3770	300	NA	0.5-0.7	Rabbit
KEAP1	KEAP1	KEAP1	Kelch-like ECH-associated protein 1		Keap1	Cell Signaling	8047	1000	10000	0.5-0.7	Rabbit
KIT	KIT	KIT	Mast/stem cell growth factor receptor Kit		kit.C	Epitomics(abcam)	1522.1	1500	18000	>0.7	Rabbit
KDM1A	KDM1A	KDM1A	Lysine Demethylase 1A		LSD1	Cell Signaling	2184	3000	20000	>0.7	Rabbit
KMT2A	KMT2A	KMT2A	Histone-lysine N-methyltransferase 2A		MLL1	cell signaling	14197	300	18000	0.5-0.7	Rabbit
KMT2D	KMT2D	KMT2D	Histone-lysine N-methyltransferase 2D		MLL2	cell signaling	63795	200	18000	>0.7	Rabbit
LATS1	LATS1	LATS1	Serine/threonine-protein kinase LATS1		LATS1	Cell Signaling	3477	500	20000	>0.7	Rabbit
LCK	LCK	LCK	Tyrosine-protein kinase Lck		LCK	Cell Signaling	2752	1000	18000	>0.7	Rabbit
LDHA	LDHA	LDHA	Lactate dehydrogenase A		LDHA	Cell Signaling	3582	250	NA	0.5-0.7	Rabbit
LEF1	LEF1	LEF1	Lymphoid enhancer-binding factor 1		LEF1	Cell Signaling	2230	2000	18000	>0.7	Rabbit
LGALS3	LGALS3	LGALS3	Galectin-3		galetin.3	Santa Cruz Biotechnology	sc32790	150	15000	>0.7	mouse
LMNB1	LMNB1	LMNB1	Lamin-B1		LaminB1	Abcam	ab33741	5000	15000	>0.7	Rabbit
LRP6.pS1490	LRP6 phospho serine 1490	LRP6	Low-density lipoprotein receptor-related protein 6	cell cycle,transcription induced	LRP6.p	Cell Signaling	2568	200	18000	>0.7	Rabbit
LYN	LYN	LYN	Tyrosine-protein kinase Lyn		Lyn	Cell Signaling	2732	400	18000	>0.7	Rabbit
MAP2K1	MAP2K1	MAP2K1	Dual specificity mitogen-activated protein kinase kinase 1		MEK1	Abcam	ab32576	20000	25000	>0.7	Rabbit
MAP2K1.2.pS217.221	MAP2K1 phospho serine 217,221	MAP2K1	Dual specificity mitogen-activated protein kinase kinase 1	cell growth altered	MEK.p	Cell Signaling	9121	2000	18000	0.5-0.7	Rabbit
MAP2K1.MAP2K2	MAP2K1.MAP2K2	MAP2K1.MAP2K2	Dual specificity mitogen-activated protein kinase kinase 1		MEK.1.2	Cell Signaling	9122	8000	18000	>0.7	Rabbit
MAP2K2	MAP2K2	MAP2K2	Dual specificity mitogen-activated protein kinase kinase 2		MEK2	Cell Signaling	9125	200	18000	>0.7	Rabbit
MAPK1	MAPK1	MAPK1	Mitogen-activated protein kinase 1		ERK2	Santa Cruz Biotechnology	sc.154	10000	18000	>0.7	Rabbit
MAPK1.3.pT202.Y204	MAPK1/3 phospho threonine 202,tyrosine 204	MAPK1	Mitogen-activated protein kinase 3	Activation	ERK.p	Cell Signaling	9101	1000	18000	>0.7	Rabbit
MAPK14	MAPK14	MAPK14	Mitogen-activated protein kinase 14		P38	Cell Signaling	9212	1000	25000	>0.7	Rabbit
MAPK14.pT180.Y182	MAPK14 phospho threonine 180,tyrosine 182	MAPK14	Mitogen-activated protein kinase 14	Activation	P38.p	Cell Signaling	9211	200	18000	>0.7	Rabbit
MAPK8.MAPK10	MAPK8.MAPK10	MAPK8.MAPK10	Mitogen-activated protein kinase 10		JNK.1.3	Santa Cruz Biotechnology	sc474	300	20000	0.5-0.7	Rabbit
MAPK8.pT183.p185	MAPK8 phospho threonine 183,185	MAPK8	Mitogen-activated protein kinase 8	Activation	JNK.p	Cell Signaling	4668	100	20000	>0.7	Rabbit
MAPK9	MAPK9	MAPK9	Mitogen-activated protein kinase 9		JNK2	Cell Signaling	4672	50	18000	0.5-0.7	Rabbit
MCL1	MCL1	MCL1	Induced myeloid leukemia cell differentiation protein Mcl-1		MCL1	Cell Signaling	5453	100	18000	>0.7	Rabbit
MDM2	MDM2	MDM2	E3 ubiquitin-protein ligase Mdm2		MDM2	Santa Cruz Biotechnology	sc813	5000	20000	0.5-0.7	Rabbit
MDM2.pS166	MDM2 phospho serine 166	MDM2	E3 ubiquitin-protein ligase Mdm2	Activation	MDM2.p	Cell Signaling	3521	300	20000	>0.7	Rabbit
MDM4	MDM4	MDM4	Protein Mdm4		MDM4.A300.257A	Cell Signaling	8000	NA	25000	>0.7	Rabbit
MEF2C	MEF2C	MEF2C	Myocyte-specific enhancer factor 2C		MEF2C	Cell Signaling	5030	500	20000	>0.7	Rabbit
MEN1	MEN1	MEN1	Menin		menin	Cell Signaling	6891	500	18000	>0.7	Rabbit
MET.pY1234.Y1235	MET phospho tyrosine 1234,1235	MET	Hepatocyte growth factor receptor	Activation	Met.c.p	Cell Signaling	3129	200	20000	>0.7	Rabbit
MMP2	MMP2	MMP2	72 kDa type IV collagenase		MMP2	Cell Signaling	4022	200	20000	>0.7	Rabbit
MNK1	MNK1	MNK1	MAP kinase-interacting serine/threonine-protein kinase 1		MNK1	Cell Signaling	2195	800	18000	>0.7	Rabbit
MSH2	MSH2	MSH2	DNA mismatch repair protein Msh2		MSH2	Cell Signaling	2850	150	25000	>0.7	mouse
MSH6	MSH6	MSH6	DNA mismatch repair protein Msh6		MSH6	Novus	22030002	2000	20000	0.5-0.7	Rabbit
MSI2	MSI2	MSI2	RNA-binding protein Musashi homolog 2		MSI2	Abcam	ab76148	1000	18000	0.5-0.7	Rabbit
MTOR	MTOR	MTOR	Serine/threonine-protein kinase mTOR		mTor	Cell Signaling	2983	500	15000	>0.7	Rabbit
MTOR.pS2448	MTOR phospho serine 2448	MTOR	Serine/threonine-protein kinase mTOR	Activation	mTOR.p	Cell Signaling	2971	100	18000	0.5-0.7	Rabbit
MUC1	MUC1	MUC1	Mucin-1		EMA	DAKO	M061329.2	400	20000	0.5-0.7	mouse
MYC	MYC	MYC	Myc proto-oncogene protein		myc	cell signaling		9402	15000	>0.7	Rabbit
MYH11	MYH11	MYH11	Myosin-11		MYH11	Novus	21370002	2000	25000	>0.7	Rabbit
MYH9.pS1943	MYH9 phospho serine 1943	MYH9	Myosin-9	cell adhesion induced	MYH9.p	Cell Signaling	5026	750	25000	>0.7	Rabbit
NCL	NCL	NCL	Nucleolin		C23	Santa Cruz Biotechnology	sc8031	200	20000	>0.7	mouse
NCSTN	NCSTN	NCSTN	Nicastrin		nicastrin	Cell Signaling	9447	400	18000	0.8469	Rabbit
NDRG1.pT346	NDRG1 phospho threonine 79	NDRG1	Protein NDRG1	Activation	NDRG.p346	Cell Signaling	3217	500	20000	>0.7	Rabbit
NDUFB4	NDUFB4	NDUFB4	NADH dehydrogenase [ubiquinone] 1 beta subcomplex subunit 4		NDUFB4	Abcam	ab110243	100	20000	>0.7	mouse
NF2	NF2	NF2	Merlin		NF2	Santa Cruz Biotechnology	sc332	800	18000	0.5017	Rabbit
NFE2L2	NFE2L2	NFE2L2	Nuclear factor erythroid 2-related factor 2		NRF2	Cell Signaling	12721	50	18000	>0.7	Rabbit
NLN	NLN	NLN	Neurolysin, mitochondrial		NLN	origene	TA504178	1000	18000	>0.7	mouse
NOL3	NOL3	NOL3	Nucleolar protein 3		ARC	Imgenex to Novus (2016)	NBP2.41753	2000	20000	0.5-0.7	Rabbit
NOTCH1	NOTCH1	NOTCH1	Neurogenic locus notch homolog protein 1		Notch1	Cell Signaling	3268	100	20000	>0.7	Rabbit
NOTCH1_cleaved	NOTCH1 cleaved	NOTCH1	Neurogenic locus notch homolog protein 1	Activation	Notch1.cle	Cell Signaling	4147	100	18000	0.5-0.7	Rabbit
NOTCH2	NOTCH2	NOTCH2	Neurogenic locus notch homolog protein 2		Notch2	Cell Signaling	4530	500	18000	>0.7	Rabbit
NOTCH3	NOTCH3	NOTCH3	Neurogenic locus notch homolog protein 3		Notch3	Santa Cruz Biotechnology	sc5593	200	18000	0.5-0.7	Rabbit
NPM1_C	NPM1_C	NPM1_C	Nucleophosmin		NPM1	Cell Signaling	3542	4000	20000	>0.7	Rabbit
NRAS	NRAS	NRAS	GTPase NRas		Ras.N	Santa Cruz Biotechnology	sc31	25	15000	>0.7	mouse
NUMB	NUMB	NUMB	Protein numb homolog		Numb	Cell Signaling	2761	1000	18000	>0.7	Rabbit
PAK1	PAK1	PAK1	Serine/threonine-protein kinase PAK 1		PAK1	Cell Signaling	2602	1000	18000	>0.7	Rabbit

PAK4	PAK4	PAK4	Serine/threonine-protein kinase PAK 4		PAK4	Cell Signaling	3242	750	18000	>0.7	Rabbit
PARK7	PARK7	PARK7	Protein/nucleic acid deglycase DJ-1		DJ.1	Dr Tek Mak	Tek	NA	18000	>0.7	rabbit
PARP	PARP	PARP	Poly [ADP-ribose] polymerase 1		PARP	Cell Signaling	9542	500	18000	0.5-0.7	Rabbit
PARP_cleaved	PARP cleaved	PARP	Poly [ADP-ribose] polymerase 1	Activation	PARP.cleavage	Cell Signaling	9541	100	20000	>0.7	Rabbit
PCNA	PCNA	PCNA	Proliferating cell nuclear antigen		PCNA	Abcam	ab29	200	18000	0.5-0.7	mouse
PDCD1	PDCD1	PDCD1	Programmed cell death protein 1		PD.1	Cell Signaling	43248	50	18000	>0.7	mouse
PDCD4	PDCD4	PDCD4	Programmed cell death protein 4		Pdcd4	Rockland	600.401.965	2000	20000	0.5-0.7	Rabbit
PDGFRB	PDGFRB	PDGFRB	Platelet-derived growth factor receptor beta		PDGFR.beta	Cell Signaling	3169	30	15000	>0.7	Rabbit
PDK1	PDK1	PDK1	vate dehydrogenase (acetyl-transferring)] kinase isozyme 1, mitochondrial		PDHK1	Cell Signaling	3820	250	15000	>0.7	Rabbit
PDK1.S241	PDK1.S241	PDK1.S241	vate dehydrogenase (acetyl-transferring)] kinase isozyme 1, mitochondrial		PDK1.p241	Cell Signaling	3061	800	15000	>0.7	Rabbit
PECAM1	PECAM1	PECAM1	Platelet endothelial cell adhesion molecule		CD31	Dako	M0823	75	15000	>0.7	mouse
PIK3CA	PIK3CA	PIK3CA	phatidylinositol 4,5-bisphosphate 3-kinase catalytic subunit alpha isoform		PI3K.alpha	Cell Signaling	4255	200	18000	0.5-0.7	Rabbit
PIK3CB	PIK3CB	PIK3CB	sphatidylinositol 4,5-bisphosphate 3-kinase catalytic subunit beta isoform		PI3K	Santa Cruz	sc376412	NA	18000	0.5-0.7	mouse
PIM1	PIM1	PIM1	Serine/threonine-protein kinase pim-1		PIM1	Santa Cruz Biotechnology	sc13513	100	20000	0.5-0.7	mouse
PIM2	PIM2	PIM2	Serine/threonine-protein kinase pim-2		PIM2	Cell Signaling	4730	75	18000	>0.7	Rabbit
PKM	PKM	PKM	Pyruvate kinase PKM		PKM2	Cell Signaling	4053	500	20000	0.5-0.7	Rabbit
PLK1	PLK1	PLK1	Serine/threonine-protein kinase PLK1		PLK1	Thermo scientific	MA5.171512	100	18000	>0.7	mouse
PPARA	PPARA	PPARA	Peroxisome proliferator-activated receptor alpha		PPAR.alpha	boster Bio	PA1412	100	18000	>0.7	Rabbit
PPARG	PPARG	PPARG	Peroxisome proliferator-activated receptor gamma		PPAR.gamma	Santa Cruz Biotechnology	sc7273	100	20000	>0.7	mouse
PREX1	PREX1	PREX1	sphatidylinositol 3,4,5-trisphosphate-dependent Rac exchanger 1 protein		PREX1	Abcam	ab102739	1000	20000	>0.7	rabbit
PRKAA1.2	PRKAA1.2	PRKAA1.2	5'-AMP-activated protein kinase catalytic subunit alpha-1		AMPK.alpha	Cell Signaling	2532	300	20000	0.5-0.7	Rabbit
PRKAA1.2.pT172	PRKAA1/2 phospho threonine 172	PRKAA1	5'-AMP-activated protein kinase catalytic subunit alpha-2	Activation	AMPK.alpha.p	Cell Signaling	2535	500	20000	0.5-0.7	Rabbit
PRKAA2.pS345	PRKAA2 phospho serine 345	PRKAA2	5'-AMP-activated protein kinase catalytic subunit alpha-2		AMPK.p345	Abcam	ab129081	500	18000	>0.7	Rabbit
PRKAR1A	PRKAR1A	PRKAR1A	cAMP-dependent protein kinase type I-alpha regulatory subunit		PKA	Cell Signaling	RI.alpha	5675	20000	>0.7	Rabbit
PRKCA	PRKCA	PRKCA	Protein kinase C alpha type		PKC.alpha	Upstate	5.154	10000	20000	>0.7	mouse
PRKCA.pS657	PRKCA phospho serine 657	PRKCA	Protein kinase C alpha type	Activation	PKC.alpha.p	Upstate	6.822	10000	18000	>0.7	Rabbit
PRKCB.pS660	PRKCB phospho serine 660	PRKCB	Protein kinase C beta type	intracellular localization	PKC.betaII	Cell Signaling	9371	500	18000	>0.7	Rabbit
PRKCD.pS664	PRKCD phospho serine 664	PRKCD	Protein kinase C delta type		PKC.delta.p	Upstate	7.875	500	20000	>0.7	Rabbit
PSMB9	PSMB9	PSMB9	Proteasome subunit beta type-9		LMP2	Abcam	ab3328	2000	18000	>0.7	Rabbit
PTEN	PTEN	PTEN	Phosphatase and tensin homolog		PTEN	Cell Signaling	9552	4000	18000	>0.7	Rabbit
PTGS2	PTGS2	PTGS2	Prostaglandin G/H synthase 2		cox.2	Cell Signaling	4842	100	20000	0.5-0.7	Rabbit
PTK2	PTK2	PTK2	Prostaglandin G/H synthase 2		FAK	Cell Signaling	3285	400	20000	>0.7	Rabbit
PTK2.pY397	PTK2 phospho tyrosine 397	PTK2	Focal adhesion kinase 1	Activation	FAK.p	Cell Signaling	3283	25	15000	>0.7	Rabbit
PTPN11	PTPN11	PTPN11	Tyrosine-protein phosphatase non-receptor type 11		SHP.2	Epitomics/abcam	1590.1	800	18000	0.5-0.7	Rabbit
PTPN11.pY542	PTPN11 phospho tyrosine 542	PTPN11	Tyrosine-protein phosphatase non-receptor type 11	Activation	SHP.2.p	Cell Signaling	3751	150	20000	0.5-0.7	Rabbit
PXN	PXN	PXN	Paxillin		paxillia	Abcam	ab32084	1000	25000	0.5-0.7	Rabbit
RAB11A.11B	RAB11A.11B	RAB11A.11B	Ras-related protein Rab-11A/Ras-related protein Rab-11B		Rab11	Cell Signaling	3539	200	18000	0.5-0.7	Rabbit
RAB25	RAB25	RAB25	Ras-related protein Rab-25		Rab25	Cell Signaling	4314	50	15000	>0.7	Rabbit

RAD50	RAD50	RAD50	DNA repair protein RAD50		Rad50	Millipore	5	525	20000	>0.7	mouse
RAD51	RAD51	RAD51	DNA repair protein RAD51 homolog 1		Rad51	Cell Signaling	8875	100	20000	>0.7	Rabbit
RAF1	RAF1	RAF1	RAF proto-oncogene serine/threonine-protein kinase		Raf.C	Millipore	4.739	300	20000	0.5-0.7	Rabbit
RB1	RB1	RB1	Retinoblastoma-associated protein		Rb	BD PharMingen	554136	500	18000	>0.7	mouse
RELAp65	RELAp65	RELAp65	Transcription factor p65		NF.KB.p65	Cell Signaling	3034	800	18000	0.5-0.7	Rabbit
RELA.pS536	RELA phospho serine 536	RELA	Transcription factor p65	Activation	NF.KB.pS536	Cell Signaling	3033	300	20000	0.5-0.7	Rabbit
RHEB	RHEB	RHEB	GTP-binding protein Rheb		Rheb	Abcam	MAB3426	150	20000	0.5-0.7	mouse
RICTOR	RICTOR	RICTOR	Rapamycin-insensitive companion of mTOR		Rictor	Cell Signaling	2114	100	20000	0.5-0.7	Rabbit
RICTOR.pT1135	RICTOR phospho threonine 1135	RICTOR	Rapamycin-insensitive companion of mTOR	Stimulate binding with 14-3-3	Rictor.p	Cell Signaling	3806	2000	18000	>0.7	Rabbit
RIPK1	RIPK1	RIPK1	Receptor-interacting serine/threonine-protein kinase 1		RIP	Cell Signaling	4926	100	NA	0.5-0.7	Rabbit
RPA2	RPA2	RPA2	Replication protein A 32 kDa subunit		RPA32	CST	2208.2	NA	NA	>0.7	Rabbit
RPA2.pS4.8	RPA2 phospho serine 4,8	RPA2	Replication protein A 32 kDa subunit		RPA32.P	Bethyl Lab	a300.245A	2000	25000	0.5-0.7	Rabbit
RPS6	RPS6	RPS6	40S ribosomal protein S6		S6RP	Cell Signaling	2217	2000	20000	>0.7	Rabbit
RPS6.pS235.236	RPS6 phospho serine 235,236	RPS6	40S ribosomal protein S6	Activation	S6RP.p235	Cell Signaling	2211	2000	18000	>0.7	Rabbit
RPS6.pS240.244	RPS6 phospho serine 240,244	RPS6	40S ribosomal protein S6	Activation	S6RP.p240	Cell Signaling	2215	1000	18000	>0.7	Rabbit
RPS6KA1.2.3	RPS6KA1.2.3	RPS6KA1.2.3	6 kinase alpha-1,Ribosomal protein S6 kinase alpha-2,Ribosomal protein S6 kinase alpha-3		RSK	Cell Signaling	9347	500	NA	NA	NA
RPS6KA1.pT573	RPS6KA1 phospho threonine 573	RPS6KA1	Ribosomal protein S6 kinase alpha-1	signaling pathway regulation	P90RSK.p573	Cell Signaling	9346	50	NA	NA	NA
RPS6KB1	RPS6KB1	RPS6KB1	Ribosomal protein S6 kinase beta-1		P70S6K	Cell Signaling	9202	500	18000	>0.7	Rabbit
RPS6KB1.pT389	RPS6KB1 phospho threonine 79	RPS6KB1	Ribosomal protein S6 kinase beta-1	Activation	P70S6K.p	Cell Signaling	9205	200	20000	>0.7	Rabbit
RPTOR	RPTOR	RPTOR	Regulatory-associated protein of mTOR		Raptor	Cell Signaling	2280	500	20000	>0.7	Rabbit
S100A4	S100A4	S100A4	S100 calcium binding protein A4		S100A4	cell signaling	13018	1000	18000	>0.7	Rabbit
SCD	SCD	SCD	Acyl-CoA desaturase		SCD	Santa Cruz Biotechnology	sc58420	100	20000	>0.7	mouse
SDHA	SDHA	SDHA	ccinate dehydrogenase [ubiquinone] flavoprotein subunit, mitochondrial		SDHA	cell signaling	11998	250	20000	>0.7	Rabbit
SETD1A	SETD1A	SETD1A	Histone-lysine N-methyltransferase SETD1A		Set1A	cell signaling	61702	400	18000	>0.7	Rabbit
SETD1B	SETD1B	SETD1B	Histone-lysine N-methyltransferase SETD1B		Set1B	cell signaling	44922	400	18000	0.5-0.7	Rabbit
SF3B1	SF3B1	SF3B1	Splicing factor 3B subunit 1		SF3B1	cell signaling	14434	2000	10000	0.5-0.7	Rabbit
SF3B1.p313	SF3B1 phospho threonine 313	SF3B1	Splicing factor 3B subunit 1		SF3B1.p313	Cell Signaling	25009	500	10000	0.5-0.7	Rabbit
SGK1	SGK1	SGK1	Serine/threonine-protein kinase Sgk1		SGK1	CST	12103	200	NA	>0.7	Rabbit
SGK3	SGK3	SGK3	Serum/glucocorticoid regulated kinase family, member 3		SGK3	Cell Signaling	8156	100	NA	>0.7	Rabbit
SHC1.pY317	SHC1 phospho tyrosine 317	SHC1	SHC-transforming protein 1		Shc.p317	Cell Signaling	2431	200	20000	>0.7	Rabbit
SIRT1	SIRT1	SIRT1	NAD-dependent protein deacetylase sirtuin-1		sirt1	Abcam	ab32441	2000	18000	0.5-0.7	Rabbit
SMAD1	SMAD1	SMAD1	Mothers against decapentaplegic homolog 1		smad1	Epitomics/abcam	1649.1	200	20000	>0.7	Rabbit
SMAD2	SMAD2	SMAD2	Mothers against decapentaplegic homolog 2		smad2	Cell Signaling	5339	5000	20000	>0.7	Rabbit
SMAD2.p245.250.255	SMAD2 phospho serine 245,250,255	SMAD2	Mothers against decapentaplegic homolog 2	Activation	smad2.p245	Cell Signaling	3104	500	20000	>0.7	Rabbit
SMAD2.pS465.467	SMAD2 phospho serine 465,467	SMAD2	Mothers against decapentaplegic homolog 2	Activation	smad2.p465.467	Cell Signaling	3108	500	20000	0.5-0.7	Rabbit
SMAD3	SMAD3	SMAD3	Mothers against decapentaplegic homolog 3		smad3	Cell Signaling	9523	500	20000	>0.7	Rabbit
SMAD4	SMAD4	SMAD4	Mothers against decapentaplegic homolog 4		smad4	Santa Cruz Biotechnology	sc7966	200	20000	>0.7	mouse
SMAD5	SMAD5	SMAD5	Mothers against decapentaplegic homolog 5		smad5	Epitomics	1682.1	1000	20000	0.5-0.7	Rabbit
SOCS2	SOCS2	SOCS2	Suppressor of cytokine signaling 2		SOCS2	Abcam	ab92847	200	20000	>0.7	Rabbit
SOD1	SOD1	SOD1	Superoxide dismutase [Cu-Zn]		SOD1	Cell Signaling	4266	4000	18000	>0.7	mouse
SOD2	SOD2	SOD2	Superoxide dismutase [Mn], mitochondrial		SOD2	cell signaling	13141	20000	25000	>0.7	Rabbit
SOX2	SOX2	SOX2	Transcription factor SOX-2		Sox2	Cell Signaling	2748	75	20000	>0.7	Rabbit
SPARC	SPARC	SPARC	SPARC		SPARC	Cell Signaling	8725	500	15000	0.5-0.7	Rabbit
SPI1	SPI1	SPI1	Transcription factor PU.1		PU1	Cell Signaling	2258	20000	20000	0.5-0.7	Rabbit
SPP1	SPP1	SPP1	Osteopontin		OPN	Santa Cruz Biotechnology	sc21742	25	15000	0.5-0.7	mouse
SQSTM1	SQSTM1	SQSTM1	Sequestosome-1		P62	Santa Cruz Biotechnology	sc28359	100	25000	0.5-0.7	mouse
SRC	SRC	SRC	Proto-oncogene tyrosine-protein kinase Src		SRC	Upstate	5.184	1000	20000	>0.7	mouse
SRC.pY527	SRC phospho tyrosine 527	SRC	Proto-oncogene tyrosine-protein kinase Src	Activation	SRC.p527	Cell Signaling	2105	400	20000	>0.7	Rabbit
SRSF1	SRSF1	SRSF1	Serine/arginine-rich splicing factor 1		SF2	invitrogen	32.45	300	25000	0.5-0.7	mouse
SSBP2	SSBP2	SSBP2	Single stranded DNA binding protein 2		SSBP2	Abcam	ab177944	1000	20000	0.5-0.7	Rabbit
STAT1	STAT1	STAT1	Signal transducer and activator of transcription 1-alpha/beta		stat1	Cell Signaling	9172	250	20000	>0.7	Rabbit
STAT3	STAT3	STAT3	Signal transducer and activator of transcription 3		stat3	Cell Signaling	4904	500	25000	0.5-0.7	Rabbit
STAT3.p705	STAT3 phospho tyrosine 705	STAT3	Signal transducer and activator of transcription 3	Activation	stat3.p705	Cell Signaling	9131	400	20000	>0.7	Rabbit
STAT3.p727	STAT3 phospho serine 727	STAT3	Signal transducer and activator of transcription 3	Activation	stat3.p727	Cell Signaling	9134	200	20000	0.5-0.7	Rabbit
STAT5A	STAT5A	STAT5A	Signal transducer and activator of transcription 5A		stat5a	Abcam	ab32043	4000	25000	>0.7	Rabbit
STK11	STK11	STK11	Serine/threonine-protein kinase STK11		LKB1	Cell Signaling	3050	500	20000	0.5-0.7	Rabbit
STK4	STK4	STK4	Serine/threonine-protein kinase 4		MST1	Cell Signaling	3682	500	20000	>0.7	Rabbit
STMN1	STMN1	STMN1	Stathmin		stathmin	Epitomics	1972.1	10000	25000	>0.7	Rabbit
SUZ12	SUZ12	SUZ12	Polycomb protein SUZ12		SUZ12	Cell Signaling	3737	200	18000	>0.7	Rabbit
SYK	SYK	SYK	Tyrosine-protein kinase SYK		Syk	Santa Cruz Biotechnology	sc1240	2000	20000	>0.7	mouse
TAPBP	TAPBP	TAPBP	Tapasin		Tapasin	Enzo life science	ADI.CSA.630.D	1000	25000	0.5-0.7	Rabbit
TAZ	TAZ	TAZ	Tafazzin		TAZ	Cell Signaling	4883	300	20000	>0.7	Rabbit
TFRC	TFRC	TFRC	Transferrin receptor protein 1		TFRC	novus	2250002	10000	25000	>0.7	Rabbit
TGM2	TGM2	TGM2	Protein-glutamine gamma-glutamyltransferase 2		TG2	Abcam	ab2386	2000	20000	>0.7	mouse
TIGAR	TIGAR	TIGAR	TP53 Induced Glycolysis Regulatory Phosphatase		tiger	Abcam	ab137573	500	20000	>0.7	Rabbit
TNFRSF4	TNFRSF4	TNFRSF4	Tumor necrosis factor receptor superfamily member 4		CD134	Abcam	ab76000	50	18000	>0.7	Rabbit
TNK1	TNK1	TNK1	Non-receptor tyrosine-protein kinase TNK1		TNK1	abgent	ap7722a	100	20000	>0.7	Rabbit
TP53	TP53	TP53	Cellular tumor antigen p53		p53	Cell Signaling	9282	2000	25000	0.5-0.7	Rabbit
TP53BP1	TP53BP1	TP53BP1	TP53-binding protein 1		X53BP1	Cell Signaling	4937	300	18000	>0.7	Rabbit
TRAP1	TRAP1	TRAP1	Heat shock protein 75 kDa, mitochondrial		HSP75	BD Biosciences	612344	750	18000	>0.7	Mouse
TSC1	TSC1	TSC1	Hamartin		TSC1	Cell Signaling	4906	400	20000	0.5-0.7	Rabbit
TSC2	TSC2	TSC2	Tuberin		TSC2	Epitomics/abcam	1613.1	500	20000	>0.7	Rabbit
TSC2.pT1462	TSC2 phospho threonine 1462	TSC2	Tuberin	Inactivation	TSC.p1462	Cell Signaling	3617	200	20000	>0.7	Rabbit
TUBA1A_Detyro	TUBA1A_Detyro	TUBA1A	Tubulin alpha-1A chain		Detyrosinated	Abcam	ab48389	500	25000	>0.7	Rabbit
TUBA4A	TUBA4A	TUBA4A	Tubulin alpha-4A chain		tubulin	Sigma	T6074	500	NA	NA	Mouse
TYRO3	TYRO3	TYRO3	Tyrosine-protein kinase receptor TYRO3		tyro3	Cell Signaling	5585.1	NA	NA	>0.7	Rabbit
UGT1A	UGT1A	UGT1A	UDP-glucuronosyltransferase 1-1		UGT1A	Santa Cruz Biotechnology	sc271268	200	NA	>0.7	mouse
ULK1.pS757	ULK1 phospho serine 757	ULK1	Serine/threonine-protein kinase ULK1	autophagy induced	ULK.p757	Cell Signaling	6888	200	18000	0.5-0.7	Rabbit
VASP	VASP	VASP	Vasodilator-stimulated phosphoprotein		VASP	Cell Signaling	3112	150	15000	>0.7	Rabbit
VCP	VCP	VCP	Transitional endoplasmic reticulum ATPase		VCP	Abcam	ab11433.200000	NA	250000	>0.7	mouse
VHL	VHL	VHL	Von Hippel-Lindau disease tumor suppressor		VHL	Novus	NB.100.485	250	20000	0.5-0.7	Rabbit
VIM	VIM	VIM	Vimentin		Vimentin	Dako	M0725	400	20000	0.5-0.7	mouse
VTCN1	VTCN1	VTCN1	V-set domain-containing T-cell activation inhibitor 1		B7.H4	cell signaling	14572	50	18000	0.5-0.7	Rabbit
WDR5	WDR5	WDR5	WD repeat-containing protein 5		WDR5	cell signaling	13105	1000	18000	>0.7	Rabbit

Wee1	Wee1	Wee1	Wee1-like protein kinase		Wee1	Cell Signaling	4936	500	20000	0.5-0.7	Rabbit
Wee1.pS642	WEE1 phospho serine 642	Wee1	Wee1-like protein kinase	Inactivation	Wee1.p	Cell Signaling	4910	50	NA	0.5-0.7	Rabbit
WTAP	WTAP	WTAP	Pre-mRNA-splicing regulator WTAP		WTAP	UTSA(Sanjay Bansal)	Sanjan	5000	20000	>0.7	Rabbit
XIAP	XIAP	XIAP	E3 ubiquitin-protein ligase XIAP		XIAP	Cell Signaling	2042	200	18000	0.5-0.7	Rabbit
XPA	XPA	XPA	DNA repair protein complementing XP-A cells		XPA	Santa Cruz Biotechnology	sc56813	75	20000	0.5-0.7	mouse
XPF	XPF	XPF	ERCC Excision Repair 4, Endonuclease Catalytic Subunit		XPF	Abcam	ab73720	50	15000	0.5-0.7	Rabbit
XPO1	XPO1	XPO1	Exportin-1		CRM1	Santa Cruz Biotechnology	sc5595	2000	20000	>0.7	Rabbit
XRCC1	XRCC1	XRCC1	DNA repair protein XRCC1		XRCC1	Cell Signaling	2735	200	20000	0.5-0.7	Rabbit
YAP	YAP	YAP	Yes1 Associated Transcriptional Regulator		YAP	Cell Signaling	4912	50	15000	0.5-0.7	Rabbit
YAP1.pS127	YAP1 phospho serine 127	YAP1	Yes1 Associated Transcriptional Regulator	Inactivation	YAP.p	Cell Signaling	4911	400	18000	0.5-0.7	Rabbit
YBX1.pS102	YBX1 phospho serine 102	YBX1	Nuclease-sensitive element-binding protein 1	Activation	YB1	Cell Signaling	2900	250	20000	>0.7	Rabbit
YWHAE	YWHAE	YWHAE	14-3-3 protein epsilon		14.3.3.epsilon	Santa Cruz Biotechnology	sc23957	200	20000	0.5-0.7	mouse
ZAP70	ZAP70	ZAP70	Tyrosine-protein kinase ZAP-70		ZAP70	Cell Signaling	2705S	300	10000	0.98	Rabbit

Supplementary Table S3

	Median HCL	Median CD19	P-value	Adjusted P-value (FDR)
AIFM1	-1.011	0.000	0.000	0.001
AKT1	-0.484	0.000	0.000	0.001
ASS1	-1.663	0.000	0.000	0.001
ATF3	-1.545	0.000	0.000	0.001
ATG3	0.884	0.000	0.000	0.001
BRAF	-1.465	0.000	0.000	0.001
CASP9	-1.331	0.000	0.000	0.001
CD4	2.572	0.000	0.000	0.001
CD74	-1.652	0.000	0.000	0.001
CDC25C	-3.095	0.000	0.000	0.001
CDK1	-1.501	0.000	0.000	0.001
CDK2	-1.528	0.000	0.000	0.001
CDKN1B	-1.034	0.000	0.000	0.001
CHEK1	-1.866	0.000	0.000	0.001
CHEK2	-1.592	0.000	0.000	0.001
DLX1	-0.628	0.000	0.000	0.001
E2F1	-3.174	0.000	0.000	0.001
EIF2AK2	-1.667	0.000	0.000	0.001
ELAVL1	-0.725	0.000	0.000	0.001
ERBB2	-1.648	0.000	0.000	0.001
ERCC1	-1.414	0.000	0.000	0.001
GATA3	-0.908	0.000	0.000	0.001
H3K4Me3	-2.172	0.000	0.000	0.001
HDAC1	-1.719	0.000	0.000	0.001
HIF1A	-1.477	0.000	0.000	0.001
HIST1H2B.Ub	-2.527	0.000	0.000	0.001
HNRNPK	-2.487	0.000	0.000	0.001
HSPA5	-1.737	0.000	0.000	0.001
ITGB1	-3.367	0.000	0.000	0.001
MAP2K1.MAP2K2	0.564	0.000	0.000	0.001
MAPK8.MAPK10	-3.193	0.000	0.000	0.001
MSH2	-1.961	0.000	0.000	0.001
MUC1	-2.208	0.000	0.000	0.001
NCL	-0.709	0.000	0.000	0.001
NCSTN	0.527	0.000	0.000	0.001
NDUFB4	-2.893	0.000	0.000	0.001
PAK1	0.821	0.000	0.000	0.001
PARK7	-1.106	0.000	0.000	0.001
PECAM1	-2.858	0.000	0.000	0.001
PIM1	-2.170	0.000	0.000	0.001
PLK1	-2.027	0.000	0.000	0.001
PRKCA.pS657	-2.125	0.000	0.000	0.001
PTGS2	-0.528	0.000	0.000	0.001
PXN	1.901	0.000	0.000	0.001

RAD50	-1.905	0.000	0.000	0.001
RHEB	-1.380	0.000	0.000	0.001
RPS6KA1.2.3	0.532	0.000	0.000	0.001
S100A4	2.693	0.000	0.000	0.001
SCD	-1.575	0.000	0.000	0.001
SMAD4	-1.257	0.000	0.000	0.001
SOCS2	-4.967	0.000	0.000	0.001
SPP1	-1.056	0.000	0.000	0.001
SQSTM1	-3.174	0.000	0.000	0.001
SRSF1	-1.337	0.000	0.000	0.001
TRAP1	-3.519	0.000	0.000	0.001
TUBA1A_Detyro	-0.861	0.000	0.000	0.001
YWHAE	-1.794	0.000	0.000	0.001
CCNE1	-1.863	0.000	0.000	0.001
JAG1	0.693	0.000	0.000	0.001
NRAS	-2.263	0.000	0.000	0.001
PCNA	-0.999	0.000	0.000	0.001
STK11	0.514	0.000	0.000	0.001
TFRC	4.836	0.000	0.000	0.001
ZAP70	2.122	0.000	0.000	0.001
CD44	-1.066	0.000	0.000	0.002
COPS5	-2.398	0.000	0.000	0.002
EEF2K	-0.502	0.000	0.000	0.002
EGFR.pY1173	-0.275	0.000	0.000	0.002
H3K27Me3	-1.051	0.000	0.000	0.002
LGALS3	-1.962	0.000	0.000	0.002
MNK1	0.656	0.000	0.000	0.002
NLN	-1.240	0.000	0.000	0.002
NOTCH3	-0.773	0.000	0.000	0.002
PDCD1	-2.187	0.000	0.000	0.002
PDGFRB	0.388	0.000	0.000	0.002
PIK3CB	-1.263	0.000	0.000	0.002
RAF1	0.468	0.000	0.000	0.002
VHL	-1.345	0.000	0.000	0.002
ANXA1	1.630	0.000	0.001	0.003
BCL2L11	-1.644	0.000	0.001	0.003
BID	1.197	0.000	0.001	0.003
CDKN1A	-0.943	0.000	0.001	0.003
DVL3	-0.215	0.000	0.001	0.003
EPHA2pS897	0.487	0.000	0.001	0.003
PKM	1.199	0.000	0.001	0.003
SGK1	0.531	0.000	0.001	0.003
SPARC	0.586	0.000	0.001	0.003
XPO1	-0.639	0.000	0.001	0.003
ATG7	0.695	0.000	0.001	0.005
FLI1	-0.690	0.000	0.001	0.005
HIST3H3	-0.467	0.000	0.001	0.005
JAK2	-0.649	0.000	0.001	0.005

JMJD6	-0.450	0.000	0.001	0.005
MAP2K2	0.411	0.000	0.001	0.005
NOTCH1_cleaved	1.580	0.000	0.001	0.005
PSMB9	-1.174	0.000	0.001	0.005
RPS6KB1	0.483	0.000	0.001	0.005
SMAD5	-3.270	0.000	0.001	0.005
SOD1	-1.137	0.000	0.001	0.005
YAP1.pS127	0.520	0.000	0.001	0.005
ACACA.pS79	0.474	0.000	0.002	0.007
BTK	0.769	0.000	0.002	0.007
CCND3	-0.537	0.000	0.002	0.007
EIF2S1.pS51	-0.438	0.000	0.002	0.007
EP300	-1.160	0.000	0.002	0.007
FOXO3	0.435	0.000	0.002	0.007
ITGAL	-1.144	0.000	0.002	0.007
ATG4B	0.983	0.000	0.003	0.009
BIRC2	-1.239	0.000	0.003	0.009
BMI1	-0.532	0.000	0.003	0.009
H3K9Me2	-0.622	0.000	0.003	0.009
HDAC2	-0.475	0.000	0.003	0.009
JUNB	0.537	0.000	0.003	0.009
PPARG	-1.532	0.000	0.003	0.009
SMAD2.p245.250.255	0.586	0.000	0.003	0.009
UGT1A	-1.143	0.000	0.003	0.009
ACTB	-0.433	0.000	0.004	0.013
BCL2	-1.285	0.000	0.004	0.013
CCND1	-0.728	0.000	0.004	0.013
MSH6	-0.528	0.000	0.004	0.013
RB1	-1.527	0.000	0.004	0.013
SMAD2	0.540	0.000	0.004	0.013
VCP	-0.479	0.000	0.004	0.013
VTCN1	0.555	0.000	0.004	0.013
YAP	0.427	0.000	0.004	0.013
AKR1C3	-0.844	0.000	0.006	0.017
ARAF	-0.346	0.000	0.006	0.017
ARID1A	-0.778	0.000	0.006	0.017
CD276	0.408	0.000	0.006	0.017
DDX17	-0.444	0.000	0.006	0.017
ERG	-0.646	0.000	0.006	0.017
LMNB1	-1.182	0.000	0.006	0.017
MDM2.pS166	-0.422	0.000	0.006	0.017
MMP2	0.408	0.000	0.006	0.017
MYH9.pS1943	0.584	0.000	0.006	0.017
NPM1_C	-0.791	0.000	0.006	0.017
RPTOR	0.597	0.000	0.006	0.017
AKT1S1.pT246	0.280	0.000	0.008	0.021
CDH2	-0.735	0.000	0.008	0.021
CSNK2A1	0.315	0.000	0.008	0.021

EPHA2.pY588	-0.454	0.000	0.008	0.021
ERBB2.pY1248	0.338	0.000	0.008	0.021
GAPDH	-1.332	0.000	0.008	0.021
GYS1.pS641	0.662	0.000	0.008	0.021
KMD1A	-0.411	0.000	0.008	0.021
MDM4	-0.264	0.000	0.008	0.021
PRKCB.pS660	-0.426	0.000	0.008	0.021
RAD51	0.313	0.000	0.008	0.021
RIPK1	0.523	0.000	0.008	0.021
TUBA4A	-0.322	0.000	0.008	0.021
ULK1.pS757	0.265	0.000	0.008	0.021
BCL2.pS70	0.312	0.000	0.011	0.026
EIF4E	0.425	0.000	0.011	0.026
HES1	0.538	0.000	0.011	0.026
JUN.pS73	-0.336	0.000	0.011	0.026
MAP2K1	0.374	0.000	0.011	0.026
PARP	-0.536	0.000	0.011	0.026
PRKAA1.2	0.551	0.000	0.011	0.026
STAT1	-0.408	0.000	0.011	0.026
TNFRSF4	0.201	0.000	0.011	0.026
WEE1.pS642	0.527	0.000	0.011	0.026
ASNS	0.404	0.000	0.015	0.033
AURKB	0.228	0.000	0.015	0.033
H2AX.p139	-1.218	0.000	0.015	0.033
H2AX.p140	-1.245	0.000	0.015	0.033
ITGA2	-0.923	0.000	0.015	0.033
PIM2	0.392	0.000	0.015	0.033
SMAD1	0.507	0.000	0.015	0.033
SMAD3	-0.278	0.000	0.015	0.033
SOX2	0.362	0.000	0.015	0.033
SRC...576	-1.160	0.000	0.015	0.033
TSC2.pT1462	0.275	0.000	0.015	0.033
ATM.pS1981	0.100	0.000	0.019	0.039
BAK	-0.371	0.000	0.019	0.039
BCL2L1	1.184	0.000	0.019	0.039
BIRC3	-0.295	0.000	0.019	0.039
CDX2	0.450	0.000	0.019	0.039
CREB...327	-0.268	0.000	0.019	0.039
DDB1	0.284	0.000	0.019	0.039
ERN1	1.048	0.000	0.019	0.039
H3K36Me3	-0.226	0.000	0.019	0.039
INPPL1	0.417	0.000	0.019	0.039
MSI2	-0.704	0.000	0.019	0.039
PRKCD.pS664	-0.430	0.000	0.019	0.039
RICTOR.pT1135	0.639	0.000	0.019	0.039
SF3B1	-0.592	0.000	0.019	0.039
SYK	-0.787	0.000	0.019	0.039
TSC1	0.678	0.000	0.019	0.039

XPF	-0.424	0.000	0.019	0.039
CASP3_cleaved	-0.234	0.000	0.025	0.048
CDKN1B.pS10	-0.740	0.000	0.025	0.048
ELK1.pS383	-0.448	0.000	0.025	0.048
GSK3A.B	-0.559	0.000	0.025	0.048
HSP90AA1.HSP90AB1	0.402	0.000	0.025	0.048
LATS1	0.214	0.000	0.025	0.048
RAB11A.11B	0.203	0.000	0.025	0.048
RICTOR	0.540	0.000	0.025	0.048
SUZ12	0.449	0.000	0.025	0.048
TIGAR	0.313	0.000	0.025	0.048
TNK1	0.173	0.000	0.025	0.048
BRD4	-0.356	0.000	0.032	0.060
PARP_cleaved	-0.719	0.000	0.032	0.060
SIRT1	-0.291	0.000	0.032	0.060
VASP	0.410	0.000	0.032	0.060
XRCC1	0.070	0.000	0.032	0.060
CHEK2.pT68	0.182	0.000	0.040	0.073
FN1	2.903	0.000	0.040	0.073
MAP2K1.2.pS217.221	0.329	0.000	0.040	0.073
NF2	-0.840	0.000	0.040	0.073
RPS6.p240.244	0.381	0.000	0.040	0.073
SF3B1.pT313	-0.418	0.000	0.040	0.073
STMN1	0.705	0.000	0.040	0.073
CREB1.pS133	-0.685	0.000	0.050	0.087
CTSG	-1.214	0.000	0.050	0.087
G6PD	0.590	0.000	0.050	0.087
GAB2.pY452	0.301	0.000	0.050	0.087
HK2	-0.690	0.000	0.050	0.087
MAPK14	0.536	0.000	0.050	0.087
PDK1.S241	0.345	0.000	0.050	0.087
PRKAA1.2.pT172	-0.505	0.000	0.050	0.087
RPA2.pS4.8	-0.454	0.000	0.050	0.087
ACACA	0.479	0.000	0.062	0.103
KEAP1	0.332	0.000	0.062	0.103
KMT2D	-0.444	0.000	0.062	0.103
NOTCH1	0.343	0.000	0.062	0.103
STAT3.pY705	0.538	0.000	0.062	0.103
TYRO3	0.226	0.000	0.062	0.103
WTAP	-0.402	0.000	0.062	0.103
XPA	-0.952	0.000	0.062	0.103
AXL	0.324	0.000	0.075	0.122
BAX	0.516	0.000	0.075	0.122
BCL2A1	-0.463	0.000	0.075	0.122
DUSP6	0.789	0.000	0.075	0.122
FOXM1	0.347	0.000	0.075	0.122
H3K4Me1	-0.391	0.000	0.075	0.122
RPS6.p235.236	0.729	0.000	0.075	0.122

EPHA2	0.165	0.000	0.091	0.143
GLS	-0.568	0.000	0.091	0.143
H3K4Me2	-0.667	0.000	0.091	0.143
NDRG1.pT346	0.221	0.000	0.091	0.143
NOL3	-0.504	0.000	0.091	0.143
SHC1.pY317	0.217	0.000	0.091	0.143
SRC.pY527	1.521	0.000	0.091	0.143
STAT5A	0.233	0.000	0.091	0.143
WEE1	-0.610	0.000	0.091	0.143
AKT1.2.3.pT308	1.337	0.000	0.109	0.166
CD86	0.212	0.000	0.109	0.166
FOXO3.pS318.S321	-0.306	0.000	0.109	0.166
FZR1	-1.167	0.000	0.109	0.166
GYS1	0.342	0.000	0.109	0.166
HSF1	-0.205	0.000	0.109	0.166
IGFBP2	0.223	0.000	0.109	0.166
KDR.pY117.5	-0.328	0.000	0.109	0.166
BRCA2	0.263	0.000	0.130	0.189
CHEK1.pS296	-0.182	0.000	0.130	0.189
IGF1R.pT1135.T1136	0.158	0.000	0.130	0.189
IGFR1	0.109	0.000	0.130	0.189
MEN1	-0.222	0.000	0.130	0.189
PRKCA	-0.688	0.000	0.130	0.189
RPS6	0.249	0.000	0.130	0.189
SGK3	0.328	0.000	0.130	0.189
SMAD2.p465.467	0.282	0.000	0.130	0.189
TP53BP1	-0.584	0.000	0.130	0.189
XIAP	0.130	0.000	0.130	0.189
CCNB1	0.684	0.000	0.153	0.218
EIF4G	0.415	0.000	0.153	0.218
HSPA9	-0.666	0.000	0.153	0.218
PTK2	0.448	0.000	0.153	0.218
RPS6KA1.pT573	0.199	0.000	0.153	0.218
ASH2L	0.267	0.000	0.179	0.248
ATM	-0.149	0.000	0.179	0.248
BECN1	-0.122	0.000	0.179	0.248
ERCC5	-0.175	0.000	0.179	0.248
ETS1	-0.566	0.000	0.179	0.248
KIT	0.496	0.000	0.179	0.248
MYH11	0.544	0.000	0.179	0.248
WDR5	-0.331	0.000	0.179	0.248
AKT1.AKT2.AKT3	0.384	0.000	0.208	0.283
CDKN1B.pT157	0.196	0.000	0.208	0.283
EZH2	-0.285	0.000	0.208	0.283
PTPN11	0.276	0.000	0.208	0.283
SPI1	-0.840	0.000	0.208	0.283
CDKN2A	0.227	0.000	0.240	0.319
DNM1L	0.613	0.000	0.240	0.319

EIF4EBP1	0.349	0.000	0.240	0.319
GAB2	0.212	0.000	0.240	0.319
PREX1	0.162	0.000	0.240	0.319
STAT3	0.271	0.000	0.240	0.319
TAZ	-0.234	0.000	0.240	0.319
BBC3	0.132	0.000	0.275	0.358
BRAF.pS445	0.102	0.000	0.275	0.358
HEXIM1	0.142	0.000	0.275	0.358
HSPB1.pS82	0.799	0.000	0.275	0.358
LRP6.pS1490	0.131	0.000	0.275	0.358
LYN	-0.421	0.000	0.275	0.358
AKT2	-0.204	0.000	0.313	0.399
BIRC5	0.132	0.000	0.313	0.399
CBX7	0.093	0.000	0.313	0.399
MAPK9	-0.632	0.000	0.313	0.399
MTOR...470	-0.226	0.000	0.313	0.399
YBX1.pS102	0.030	0.000	0.313	0.399
CDH1	-0.046	0.000	0.354	0.438
CDKN1B.pT198	-0.207	0.000	0.354	0.438
LDHA	0.296	0.000	0.354	0.438
MAPK1	0.102	0.000	0.354	0.438
MYC	0.166	0.000	0.354	0.438
PPARA	-0.078	0.000	0.354	0.438
RELA.pS536	-0.244	0.000	0.354	0.438
SETD1B	-0.149	0.000	0.354	0.438
SOD2	0.352	0.000	0.354	0.438
AKT3	-0.042	0.000	0.398	0.479
ANXA7	-0.164	0.000	0.398	0.479
CASP3	0.257	0.000	0.398	0.479
CTNNB1.pS33.S37.T41	-0.299	0.000	0.398	0.479
DUSP4	0.106	0.000	0.398	0.479
EGLN1	-0.082	0.000	0.398	0.479
FASN	0.374	0.000	0.398	0.479
KMT2A	-0.116	0.000	0.398	0.479
TGM2	0.553	0.000	0.398	0.479
EIF4EBP1.pS65	0.137	0.000	0.445	0.528
KDR	0.137	0.000	0.445	0.528
NFE2L2	0.168	0.000	0.445	0.528
PRKAR1A	0.222	0.000	0.445	0.528
RELA	0.216	0.000	0.445	0.528
CBL	-0.250	0.000	0.495	0.572
EGFR	0.024	0.000	0.495	0.572
EIF4G2	-0.340	0.000	0.495	0.572
GLUD	0.149	0.000	0.495	0.572
MET.pY1234.Y1235	0.016	0.000	0.495	0.572
PAK4	0.007	0.000	0.495	0.572
PTPN11.pY542	0.296	0.000	0.495	0.572
TSC2	-0.106	0.000	0.495	0.572

EZRIN	0.099	0.000	0.548	0.626
MCL1	-0.128	0.000	0.548	0.626
PTEN	-0.185	0.000	0.548	0.626
STAT3.pS727	0.197	0.000	0.548	0.626
CTNNB1	-0.132	0.000	0.603	0.681
DNMT1	0.119	0.000	0.603	0.681
HDAC3	0.037	0.000	0.603	0.681
SSBP2	0.157	0.000	0.603	0.681
AKT1.2.3.pS473	0.082	0.000	0.660	0.732
CAV1	-0.305	0.000	0.660	0.732
EIF2S1	0.037	0.000	0.660	0.732
EIF4E.pS209	-0.100	0.000	0.660	0.732
HSPD1	0.292	0.000	0.660	0.732
MAPK14.pT180.Y182	0.336	0.000	0.660	0.732
CXCR5	0.240	0.000	0.719	0.782
KAT2A	0.022	0.000	0.719	0.782
LEF1	0.159	0.000	0.719	0.782
MDM2	0.102	0.000	0.719	0.782
NOTCH2	-0.330	0.000	0.719	0.782
PRKAA2.pS345	0.066	0.000	0.719	0.782
RPS6KB1.pT389	-0.137	0.000	0.719	0.782
CASP7_cleaved	-0.019	0.000	0.780	0.818
CDK1.pY15	-0.039	0.000	0.780	0.818
CHEK1.pS345	0.017	0.000	0.780	0.818
CLPP	-0.077	0.000	0.780	0.818
COG3	-0.064	0.000	0.780	0.818
CTNNB1.pT41.S45	0.007	0.000	0.780	0.818
LCK	0.226	0.000	0.780	0.818
MAPK1.3.pT202.Y204	-0.031	0.000	0.780	0.818
MEF2C	-0.165	0.000	0.780	0.818
MTOR.pS2448	-0.166	0.000	0.780	0.818
SDHA	0.067	0.000	0.780	0.818
STK4	-0.100	0.000	0.780	0.818
TP53...597	-0.132	0.000	0.780	0.818
HSF1.pS326	-0.052	0.000	0.842	0.871
MAPK8.pT183.p185	-0.186	0.000	0.842	0.871
NUMB	0.012	0.000	0.842	0.871
PDK1	0.090	0.000	0.842	0.871
RPA2	0.060	0.000	0.842	0.871
GATA1	-0.030	0.000	0.905	0.924
GSK3A.B.pS12.9	0.115	0.000	0.905	0.924
HSPA1A	0.364	0.000	0.905	0.924
PIK3CA	0.045	0.000	0.905	0.924
SETD1A	-0.005	0.000	0.905	0.924
ABL1	-0.078	0.000	0.968	0.978
EEF2	0.142	0.000	0.968	0.978
HDAC6	0.119	0.000	0.968	0.978
PTK2.pY397	0.113	0.000	0.968	0.978

PDCD4	0.081	0.000	1.000	1.000
RAB25	0.011	0.000	1.000	1.000
TAPBP	-0.021	0.000	1.000	1.000
VIM	0.161	0.000	1.000	1.000

Supplementary table S4

Protein	Median HCL-c (n=12)	Median HCL-v (n=4)	P-value	P (FDR)
PDK1.S241	0.271	0.984	0.001	0.211
BIRC5	0.204	-0.251	0.002	0.211
E2F1	-3.053	-3.594	0.002	0.211
STK11	0.579	0.210	0.002	0.211
EIF4EBP1.pS65	-0.030	0.980	0.004	0.227
RPS6.p235.236	0.510	1.526	0.004	0.227
CD44	-0.880	-1.344	0.008	0.227
CDKN1B.pT198	-0.436	0.261	0.008	0.227
CREB1.pS133	-0.921	0.014	0.008	0.227
PTK2.pY397	0.277	-0.260	0.008	0.227
SETD1A	-0.479	0.262	0.008	0.227
SPI1	-1.212	0.668	0.008	0.227
BCL2L1	1.241	-0.055	0.008	0.810
ATG3	1.038	0.494	0.013	0.281
BCL2L11	-2.057	-1.177	0.013	0.281
CBL	-0.352	0.710	0.013	0.281
PIM2	0.506	0.144	0.013	0.281
TSC2	-0.308	0.541	0.013	0.281
ANXA7	-0.094	-1.157	0.020	0.281
AURKB	0.321	0.104	0.020	0.281
CDK1	-1.674	-0.995	0.020	0.281
CDKN2A	1.085	-0.445	0.020	0.281
KMD1A	-0.544	-0.076	0.020	0.281
NLN	-1.379	-0.564	0.020	0.281
TFRC	5.958	1.457	0.020	0.281
WTAP	-0.486	-0.127	0.020	0.281
YAP	0.615	0.136	0.020	0.281
CHEK2	-1.646	-1.335	0.030	0.316
ERBB2.pY1248	0.431	0.248	0.030	0.316
ERN1	1.676	0.589	0.030	0.316
FOXO3	0.594	0.129	0.030	0.316
H3K27Me3	-1.286	-0.738	0.030	0.316
LEF1	0.589	-0.665	0.030	0.316
PARK7	-1.010	-1.284	0.030	0.316
SMAD2.p245.250.255	0.689	0.311	0.030	0.316
SYK	-1.292	-0.099	0.030	0.316
BTK	1.103	0.331	0.042	0.341
EZRIN	0.002	0.842	0.042	0.341
HSPA1A	0.633	-0.612	0.042	0.341
MAPK8.MAPK10	-3.117	-3.371	0.042	0.341
MCL1	-0.380	0.188	0.042	0.341
PAK1	0.664	1.266	0.042	0.341
PDCD4	-0.194	1.092	0.042	0.341
PLK1	-1.942	-2.198	0.042	0.341

RAB11A.11B	0.275	-0.035	0.042	0.341
SF3B1.pT313	-0.442	-0.064	0.042	0.341
TNFRSF4	0.263	0.073	0.042	0.341
H2AX.p139	-1.330	-0.969	0.058	0.367
H3K9Me2	-0.827	-0.304	0.058	0.367
ITGB1	-3.278	-3.603	0.058	0.367
KMT2A	-0.206	0.223	0.058	0.367
PDGFRB	0.425	0.253	0.058	0.367
PKM	1.011	1.893	0.058	0.367
SGK3	-0.026	0.482	0.058	0.367
SMAD2	0.617	0.254	0.058	0.367
SMAD4	-1.245	-1.400	0.058	0.367
SOX2	0.569	0.234	0.058	0.367
SQSTM1	-2.973	-3.304	0.058	0.367
SRSF1	-1.453	-1.032	0.058	0.367
STK4	-0.202	0.130	0.058	0.367
WEE1	-0.812	0.361	0.058	0.367
BRAF.pS445	0.050	0.408	0.078	0.375
CDX2	0.502	0.095	0.078	0.375
CREB...327	-0.312	-0.071	0.078	0.375
ERBB2	-1.590	-1.864	0.078	0.375
H3K36Me3	-0.311	-0.050	0.078	0.375
KAT2A	-0.350	0.156	0.078	0.375
LCK	0.432	-0.407	0.078	0.375
MAP2K1	0.347	0.514	0.078	0.375
NF2	-0.937	-0.352	0.078	0.375
NOTCH1_cleaved	2.144	0.432	0.078	0.375
PREX1	0.145	0.894	0.078	0.375
PRKCB.pS660	-0.468	-0.187	0.078	0.375
RAF1	0.506	0.378	0.078	0.375
SETD1B	-0.253	0.178	0.078	0.375
SF3B1	-0.690	-0.126	0.078	0.375
SPARC	0.837	0.312	0.078	0.375
SSBP2	-0.081	0.961	0.078	0.375
TGM2	0.773	-0.460	0.078	0.375
VTCN1	0.659	0.323	0.078	0.375
ACACA	0.626	0.112	0.103	0.422
AKT1.2.3.pS473	0.149	-0.349	0.103	0.422
ASNS	0.688	0.216	0.103	0.422
CHEK1	-1.794	-2.111	0.103	0.422
CTSG	-1.283	0.350	0.103	0.422
DNM1L	0.642	-0.029	0.103	0.422
HEXIM1	0.060	0.471	0.103	0.422
ITGA2	-0.759	-1.095	0.103	0.422
JUNB	0.597	0.182	0.103	0.422
MSI2	-0.360	-1.475	0.103	0.422
PTGS2	-0.548	-0.461	0.103	0.422
RB1	-1.648	-0.953	0.103	0.422

RPA2.pS4.8	-0.625	-0.142	0.103	0.422
TIGAR	0.371	0.117	0.103	0.422
ACACA.pS79	0.481	0.260	0.133	0.452
AKT1	-0.562	-0.343	0.133	0.452
AKT1S1.pT246	0.326	0.051	0.133	0.452
ATG4B	1.041	0.409	0.133	0.452
BBC3	0.048	0.314	0.133	0.452
BIRC3	-0.223	-0.509	0.133	0.452
CD74	-1.718	-1.449	0.133	0.452
CDC25C	-2.971	-3.266	0.133	0.452
EEF2K	-0.523	-0.324	0.133	0.452
EIF4G2	-0.100	-0.692	0.133	0.452
HSF1.pS326	-0.180	0.428	0.133	0.452
IGFR1	0.041	0.261	0.133	0.452
KDR	0.235	-0.078	0.133	0.452
MEN1	-0.248	-0.141	0.133	0.452
PDK1	0.155	-0.307	0.133	0.452
PECAM1	-2.679	-3.016	0.133	0.452
PRKAR1A	0.149	0.703	0.133	0.452
SPP1	-0.927	-1.223	0.133	0.452
YBX1.pS102	-0.007	0.154	0.133	0.452
CSNK2A1	0.353	0.213	0.170	0.519
EIF2S1	0.164	-0.155	0.170	0.519
FASN	0.611	-0.116	0.170	0.519
FLI1	-0.922	-0.645	0.170	0.519
H3K4Me1	-0.418	-0.157	0.170	0.519
HDAC2	-0.580	-0.320	0.170	0.519
HDAC3	0.071	-0.034	0.170	0.519
HSF1	-0.236	-0.083	0.170	0.519
MAPK14	0.457	1.025	0.170	0.519
MDM2.pS166	-0.518	-0.210	0.170	0.519
MSH2	-1.944	-2.005	0.170	0.519
SMAD1	0.716	0.226	0.170	0.519
STAT1	-0.388	-0.634	0.170	0.519
ANXA1	1.659	1.223	0.212	0.566
DDB1	0.304	0.128	0.212	0.566
DVL3	-0.247	-0.160	0.212	0.566
EGFR	0.101	-0.103	0.212	0.566
HDAC1	-1.836	-1.553	0.212	0.566
HIF1A	-1.367	-1.590	0.212	0.566
HIST1H2B.Ub	-2.482	-2.658	0.212	0.566
IGFBP2	0.250	0.046	0.212	0.566
LMNB1	-1.587	-0.876	0.212	0.566
LYN	-0.362	-0.615	0.212	0.566
NOL3	-0.506	-0.189	0.212	0.566
PTPN11.pY542	0.490	-0.164	0.212	0.566
RICTOR	0.574	0.046	0.212	0.566
RPS6KB1.pT389	-0.196	0.212	0.212	0.566

STAT5A	0.195	0.634	0.212	0.566
ULK1.pS757	0.309	0.028	0.212	0.566
VHL	-1.326	-1.607	0.212	0.566
XPF	-0.568	-0.287	0.212	0.566
ASH2L	0.203	0.407	0.262	0.601
ATG7	0.763	0.395	0.262	0.601
CASP9	-1.232	-1.648	0.262	0.601
CAV1	-0.190	-0.563	0.262	0.601
EIF4E	0.474	0.275	0.262	0.601
FZR1	-1.137	-1.433	0.262	0.601
GAB2.pY452	0.393	0.176	0.262	0.601
H2AX.p140	-1.336	-1.073	0.262	0.601
HIST3H3	-0.553	-0.352	0.262	0.601
JAK2	-1.084	-0.463	0.262	0.601
KDR.pY117.5	-0.381	-0.067	0.262	0.601
LRP6.pS1490	0.245	0.044	0.262	0.601
MMP2	0.496	0.362	0.262	0.601
MTOR.pS2448	-0.191	0.059	0.262	0.601
MYH9.pS1943	0.628	0.335	0.262	0.601
NRAS	-2.282	-2.149	0.262	0.601
PAK4	-0.010	0.031	0.262	0.601
PRKCA	-0.523	-1.094	0.262	0.601
RICTOR.pT1135	0.755	0.185	0.262	0.601
RPTOR	0.641	0.246	0.262	0.601
SDHA	0.223	-0.113	0.262	0.601
SRC...576	-1.105	-1.670	0.262	0.601
TSC1	0.720	0.585	0.262	0.601
BCL2	-1.079	-1.625	0.316	0.227
AKT1.2.3.pT308	0.955	2.636	0.316	0.643
AXL	0.505	0.156	0.316	0.643
BRCA2	0.270	-0.080	0.316	0.643
CASP7_cleaved	0.124	-0.118	0.316	0.643
CCND1	-0.700	-0.796	0.316	0.643
CDK1.pY15	0.102	-0.149	0.316	0.643
EP300	-1.250	-0.781	0.316	0.643
GYS1	0.266	0.733	0.316	0.643
H3K4Me3	-2.308	-2.068	0.316	0.643
IGF1R.pT1135.T1136	0.227	0.123	0.316	0.643
JAG1	0.713	0.566	0.316	0.643
MAPK1	0.229	-0.199	0.316	0.643
MNK1	0.738	0.415	0.316	0.643
MTOR...470	-0.095	-0.597	0.316	0.643
PIK3CA	0.312	-0.172	0.316	0.643
PPARG	-1.690	-1.184	0.316	0.643
RPS6	0.213	0.413	0.316	0.643
SHC1.pY317	0.294	0.058	0.316	0.643
TRAP1	-3.481	-4.126	0.316	0.643
WEE1.pS642	0.460	0.558	0.316	0.643

XIAP	0.130	0.048	0.316	0.643
AKT2	-0.316	0.061	0.379	0.693
ARAF	-0.346	-0.208	0.379	0.693
BAK	-0.335	-0.551	0.379	0.693
FOXM1	0.377	0.199	0.379	0.693
GAPDH	-1.265	-1.966	0.379	0.693
HES1	0.467	0.646	0.379	0.693
KMT2D	-0.587	-0.278	0.379	0.693
MAPK1.3.pT202.Y204	-0.138	0.558	0.379	0.693
NUMB	-0.016	0.354	0.379	0.693
PIM1	-2.194	-1.971	0.379	0.693
PPARA	-0.058	-0.113	0.379	0.693
PTPN11	0.417	0.047	0.379	0.693
RAD51	0.368	0.197	0.379	0.693
RPS6KB1	0.519	0.445	0.379	0.693
SOCS2	-4.933	-5.071	0.379	0.693
SOD1	-1.131	-1.368	0.379	0.693
TAPBP	-0.092	0.113	0.379	0.693
TP53...597	-0.086	-0.260	0.379	0.693
TSC2.pT1462	0.312	0.170	0.379	0.693
VCP	-0.524	-0.276	0.379	0.693
XPO1	-0.680	-0.475	0.379	0.693
BCL2.pS70	0.350	0.234	0.446	0.643
ATF3	-1.545	-1.590	0.446	0.742
BMI1	-0.476	-0.626	0.446	0.742
CD276	0.464	0.294	0.446	0.742
CDKN1A	-0.910	-1.819	0.446	0.742
CDKN1B	-0.899	-1.161	0.446	0.742
CHEK1.pS345	-0.038	0.110	0.446	0.742
ERG	-0.760	-0.424	0.446	0.742
GAB2	0.288	0.144	0.446	0.742
GSK3A.B	-0.445	-1.106	0.446	0.742
MAP2K2	0.458	0.388	0.446	0.742
MAPK8.pT183.p185	-0.262	0.140	0.446	0.742
MYC	0.172	-0.016	0.446	0.742
NCSTN	0.586	0.422	0.446	0.742
NOTCH1	0.386	0.230	0.446	0.742
NOTCH3	-0.806	-0.609	0.446	0.742
PARP_cleaved	-0.634	-0.856	0.446	0.742
PRKCA.pS657	-1.893	-2.342	0.446	0.742
RHEB	-1.290	-1.434	0.446	0.742
XPA	-0.798	-1.057	0.446	0.742
XRCC1	0.083	0.055	0.446	0.742
ASS1	-1.577	-1.782	0.521	0.781
CCNB1	0.809	-0.118	0.521	0.781
CD4	3.001	2.214	0.521	0.781
CDKN1B.pT157	0.196	0.148	0.521	0.781
CLPP	0.055	-0.246	0.521	0.781

DUSP6	0.833	0.634	0.521	0.781
ERCC5	-0.125	-0.480	0.521	0.781
GLUD	0.256	-0.189	0.521	0.781
GYS1.pS641	0.610	0.903	0.521	0.781
H3K4Me2	-0.667	-0.663	0.521	0.781
HDAC6	0.378	-0.283	0.521	0.781
HSPA5	-1.737	-1.793	0.521	0.781
LGALS3	-2.048	-1.781	0.521	0.781
MUC1	-2.113	-2.281	0.521	0.781
NFE2L2	0.194	0.048	0.521	0.781
NPM1_C	-0.833	-0.791	0.521	0.781
PRKAA1.2.pT172	-0.534	-0.311	0.521	0.781
PRKAA2.pS345	-0.004	0.135	0.521	0.781
PTEN	-0.185	-0.305	0.521	0.781
RAD50	-1.949	-1.872	0.521	0.781
SGK1	0.560	0.521	0.521	0.781
SMAD2.p465.467	0.282	0.224	0.521	0.781
STAT3.pY705	0.563	0.384	0.521	0.781
STMN1	0.872	0.604	0.521	0.781
UGT1A	-1.129	-1.323	0.521	0.781
BCL2A1	-0.377	-0.545	0.599	0.742
AKT3	-0.028	-0.139	0.599	0.810
BAX	0.599	0.332	0.599	0.810
BRAF	-1.484	-1.429	0.599	0.810
CCNE1	-1.885	-1.813	0.599	0.810
CDH2	-0.783	-0.602	0.599	0.810
CHEK1.pS296	-0.189	-0.121	0.599	0.810
COG3	-0.091	0.075	0.599	0.810
CTNNB1	-0.132	-0.250	0.599	0.810
CTNNB1.pT41.S45	0.007	-0.054	0.599	0.810
EEF2	0.100	0.331	0.599	0.810
EGLN1	-0.082	-0.060	0.599	0.810
EIF2AK2	-1.743	-1.553	0.599	0.810
EIF2S1.pS51	-0.438	-0.516	0.599	0.810
EIF4E.pS209	-0.060	-0.314	0.599	0.810
EIF4G	0.486	0.383	0.599	0.810
HSPD1	0.170	0.448	0.599	0.810
INPPL1	0.417	0.421	0.599	0.810
MAPK14.pT180.Y182	0.184	0.793	0.599	0.810
PCNA	-1.044	-0.905	0.599	0.810
PXN	2.053	1.804	0.599	0.810
RELA	0.216	0.244	0.599	0.810
RELA.pS536	-0.394	-0.067	0.599	0.810
RIPK1	0.554	0.347	0.599	0.810
VIM	0.190	-0.253	0.599	0.810
WDR5	-0.347	-0.129	0.599	0.810
YAP1.pS127	0.520	0.887	0.599	0.810
ZAP70	2.122	1.712	0.599	0.810

ACTB	-0.433	-0.389	0.684	0.852
AIFM1	-0.988	-1.033	0.684	0.852
ARID1A	-0.789	-0.767	0.684	0.852
BECN1	-0.122	-0.151	0.684	0.852
BID	1.197	1.250	0.684	0.852
BRD4	-0.334	-0.560	0.684	0.852
COPS5	-2.398	-2.462	0.684	0.852
DUSP4	0.096	0.131	0.684	0.852
EPHA2	0.152	0.165	0.684	0.852
EPHA2.pY588	-0.454	-0.497	0.684	0.852
EZH2	-0.257	-0.384	0.684	0.852
FOXO3.pS318.S321	-0.196	-0.514	0.684	0.852
GATA1	-0.088	0.011	0.684	0.852
GSK3A.B.pS12.9	0.115	0.192	0.684	0.852
HSP90AA1.HSP90AB1	0.347	0.402	0.684	0.852
KEAP1	0.372	0.219	0.684	0.852
LDHA	0.331	0.253	0.684	0.852
MEF2C	-0.229	0.185	0.684	0.852
MSH6	-0.568	-0.451	0.684	0.852
NCL	-0.705	-0.789	0.684	0.852
PRKAA1.2	0.551	0.692	0.684	0.852
PSMB9	-1.129	-1.320	0.684	0.852
SMAD3	-0.275	-0.328	0.684	0.852
VASP	0.390	0.435	0.684	0.852
AKR1C3	-0.773	-0.881	0.770	0.894
AKT1.AKT2.AKT3	0.384	0.355	0.770	0.894
BIRC2	-1.249	-1.091	0.770	0.894
CD86	0.212	0.213	0.770	0.894
CTNNB1.pS33.S37.T41	-0.300	-0.189	0.770	0.894
DDX17	-0.444	-0.343	0.770	0.894
DLX1	-0.628	-0.618	0.770	0.894
FN1	2.903	2.574	0.770	0.894
GLS	-0.536	-0.661	0.770	0.894
HK2	-0.690	-0.507	0.770	0.894
KIT	0.496	0.535	0.770	0.894
MDM2	0.085	0.163	0.770	0.894
MET.pY1234.Y1235	0.011	0.016	0.770	0.894
PTK2	0.448	0.442	0.770	0.894
RAB25	-0.020	0.028	0.770	0.894
RPA2	-0.060	0.211	0.770	0.894
RPS6.p240.244	0.381	0.436	0.770	0.894
RPS6KA1.pT573	0.199	0.416	0.770	0.894
SMAD5	-3.140	-3.270	0.770	0.894
STAT3	0.271	0.319	0.770	0.894
STAT3.pS727	0.197	0.153	0.770	0.894
TNK1	0.173	0.185	0.770	0.894
TP53BP1	-0.584	-0.524	0.770	0.894
ATM	-0.149	0.084	0.862	0.935

ATM.pS1981	0.087	0.151	0.862	0.935
CASP3	0.190	0.546	0.862	0.935
CBX7	0.108	0.064	0.862	0.935
CDH1	-0.046	-0.041	0.862	0.935
CDKN1B.pS10	-0.740	-0.674	0.862	0.935
CXCR5	0.240	0.163	0.862	0.935
ELK1.pS383	-0.448	-0.493	0.862	0.935
G6PD	0.590	0.442	0.862	0.935
HSPA9	-0.588	-0.834	0.862	0.935
JUN.pS73	-0.336	-0.378	0.862	0.935
MAP2K1.MAP2K2	0.573	0.465	0.862	0.935
MAPK9	-0.558	-0.692	0.862	0.935
MDM4	-0.231	-0.294	0.862	0.935
NDUFB4	-2.863	-2.896	0.862	0.935
NOTCH2	-0.200	-0.383	0.862	0.935
PIK3CB	-1.277	-1.263	0.862	0.935
PRKCD.pS664	-0.430	-0.390	0.862	0.935
RPS6KA1.2.3	0.507	0.627	0.862	0.935
SOD2	0.377	0.217	0.862	0.935
SUZ12	0.449	0.449	0.862	0.935
TUBA4A	-0.299	-0.394	0.862	0.935
TYRO3	0.262	0.182	0.862	0.935
CASP3_cleaved	-0.234	-0.205	0.953	0.981
CCND3	-0.537	-0.452	0.953	0.981
CHEK2.pT68	0.182	0.194	0.953	0.981
DNMT1	0.119	-0.057	0.953	0.981
EIF4EBP1	0.314	0.422	0.953	0.981
HSPB1.pS82	0.799	1.252	0.953	0.981
LATS1	0.201	0.315	0.953	0.981
NDRG1.pT346	0.221	0.347	0.953	0.981
PARP	-0.536	-0.498	0.953	0.981
S100A4	2.693	2.512	0.953	0.981
SIRT1	-0.291	-0.310	0.953	0.981
YWHAE	-1.794	-1.786	0.953	0.981
CDK2	-1.537	-1.503	0.953	0.981
EGFR.pY1173	-0.281	-0.264	0.953	0.981
GATA3	-0.924	-0.906	0.953	0.981
MYH11	0.544	0.461	0.953	0.981
PDCD1	-2.187	-2.183	0.953	0.981
TAZ	-0.234	-0.196	0.953	0.981
TUBA1A_Detyro	-0.861	-0.807	0.953	0.981
ABL1	-0.078	-0.085	1.000	1.000
ELAVL1	-0.725	-0.696	1.000	1.000
EPHA2pS897	0.487	0.489	1.000	1.000
ERCC1	-1.408	-1.414	1.000	1.000
ETS1	-0.598	-0.566	1.000	1.000
HNRNPK	-2.487	-2.466	1.000	1.000
ITGAL	-1.131	-1.179	1.000	1.000

JMJD6	-0.463	-0.402	1.000	1.000
MAP2K1.2.pS217.221	0.244	0.518	1.000	1.000
SCD	-1.575	-1.568	1.000	1.000
SRC.pY527	1.396	1.544	1.000	1.000
